# Modeling neurodegenerative diseases with brain organoids: from development to disease applications

**DOI:** 10.3389/fcell.2025.1663286

**Published:** 2025-11-06

**Authors:** Teresa Larriba-González, Marina García-Martín, Doddy Denise Ojeda-Hernández, Paula Rincón-Cerrada, Lucía Martín-Blanco, María Soledad Benito-Martín, Belén Selma-Calvo, Sarah de la Fuente-Martín, Jordi A Matias-Guiu, Jorge Matias-Guiu, Ulises Gómez-Pinedo

**Affiliations:** 1 Laboratory of Neurobiology and Advanced Therapies, Institute of Neurosciences, San Carlos Health Research Institute (IdISSC), Department of Neurology, San Carlos Clinical Hospital, Madrid, Spain; 2 Institute of Neurosciences, San Carlos Health Research Institute (IdISSC), Department of Neurology, San Carlos Clinical Hospital, Complutense University of Madrid, Madrid, Spain

**Keywords:** brain organoids, neurodegenerative diseases, stem cells, applications, disease modeling, personalized medicine, regenerative medicine

## Abstract

Organoids derived from stem cells have significantly advanced disease modeling, particularly in neurodegenerative disorders, while advancing personalized and regenerative medicine. These three-dimensional structures reproduce key aspects of human brain organization and functionality, while remaining simplified models that do not yet recapitulate full neural circuitry or disease progression, providing an improved platform for studying disease mechanisms, drug responses, and potential therapeutic strategies. This review explores the methodologies used in organoid development, including the differentiation of stem cells and culture techniques that enable the formation of self-organizing tissues. Organoids have been successfully used to model key cellular and molecular aspects of neurodegenerative diseases such as Alzheimer’s and Parkinson’s, offering insights into early disease mechanisms and potential novel treatment strategies. Key findings highlight that organoids provide more physiologically relevant data than traditional two-dimensional cultures and animal models, making them valuable tools for preclinical research and personalized treatment approaches. However, challenges remain, including variability in organoid generation, lack of vascularization, and difficulties in large-scale production for clinical applications. For the effective integration of organoids into biomedical and clinical applications, future research should prioritize improving reproducibility, standardization, and vascularization methods. Addressing these limitations will enhance their translational potential, leading to more effective treatments for neurodegenerative disorders and broader applications in precision medicine.

## Graphical Abstract

**Figure:**
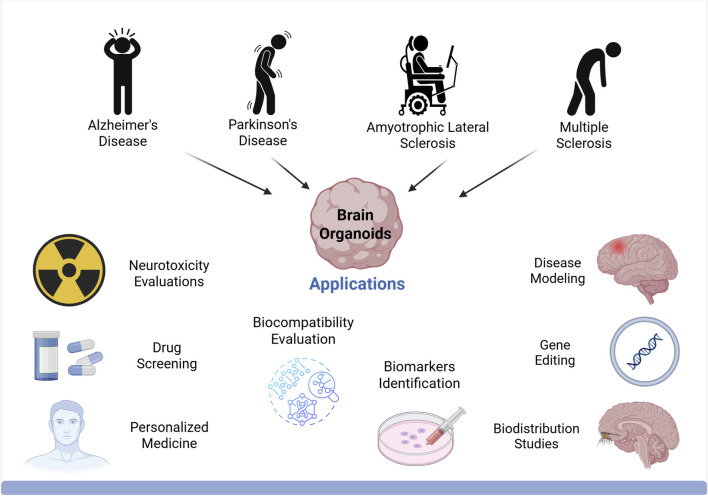
Created in BioRender. Gomez Pinedo, U. (2025) https://BioRender.com/c3mmsqg.

## 1 Introduction

Neurodegenerative diseases (NDDs) arise from the progressive loss of neurons, brain function, and cognition, affecting millions worldwide. Despite extensive research, no treatments currently halt or slow neurodegeneration. Advancing effective therapies requires a deeper understanding of brain complexity, NDD pathophysiology, and reliable *in vitro* drug-screening models. While animal models have enhanced our understanding of disease mechanisms, their translational success remains limited (Chang et al., 2020). Only about 5% of preclinical studies in animal models ultimately lead to regulatory approval for human use. For example, in diseases like Alzheimer’s (AD) and multiple sclerosis (MS), many therapies showing promise in preclinical stages fail to translate clinically (Ineichen et al., 2024). This gap stems from key differences in brain development (Quadrato et al., 2016), architecture, immune responses (Bjornson-Hooper et al., 2022), and metabolism between humans and animals, complicating cross-species extrapolation (Kuzawa et al., 2014).

Although human brain tissue is considered the gold standard for NDD research, its use is restricted by ethical and practical challenges, such as limited availability, sample sharing issues, and preservation difficulties (Quadrato et al., 2016). Post-mortem tissue also suffers from irreversible changes that may alter results, limiting its suitability for large-scale studies (Chang et al., 2020). These limitations highlight the urgent need for alternative models that accurately replicate human brain structure and function while being more ethically viable and accessible.

One promising solution is the use of three-dimensional (3D) organoids. Over the past few years, 3D organoid technology has gained significant relevance in NDD research. These organoids are small, *in vitro* 3D structures derived from human pluripotent stem cells (PSCs), such as embryonic stem cells (ESCs) and induced pluripotent stem cells (iPSCs) (Clevers, 2016; D’Antoni et al., 2023), or from adult stem cells (ASCs) (Clevers, 2016).

Since organoids are generated from human stem cells, which have the capacity to self-organize, they can recapitulate several aspects of the composition, organization and function of organs *in vivo* (Clevers, 2016), although they still lack full physiological complexity. Organoids retain key phenotypic traits of the original tissue, including a degree of cellular diversity and cell-to-cell interactions, and they have a long lifespan in culture (Quadrato et al., 2017; Trujillo et al., 2019). Furthermore, neurons within organoids have been shown to exhibit signs of polarity, migration (Clevers, 2016) and electrical activity (Quadrato et al., 2017; Trujillo et al., 2019). These characteristics make organoids a powerful platform for investigating selected cellular and molecular mechanisms of the organs they model.

Human brain organoids not only feature the development of neurons, but also glial cells, with transcriptional profiles and neurodevelopmental trajectories that closely resemble fetal brain development (Trujillo et al., 2019). This makes them a powerful tool for studying the patterning and specification of various neuronal and glial cell types. Therefore, while organoids can recapitulate important aspects of the structure and function of the human brain, they represent a simplified system that captures early developmental and disease-related processes, offering unique opportunities to explore features of human brain biology that cannot be effectively modeled in animals, and holding particular promise for studying neurological and neurodegenerative disorders (D’Antoni et al., 2023).

This review aims to provide a broad, up-to-date evaluation of the use of brain organoids in the study of NDDs and their potential applications in precision medicine. It is intended as a conceptual synthesis of different organoid models, addressing the main advances, opportunities, challenges and future perspectives in this rapidly evolving field. With this approach, we provide a comprehensive yet accessible resource for researchers and clinicians seeking to better understand the mechanisms of neurodegeneration and to advance the development of personalized therapeutic strategies.

## 2 The evolution of 3D cell cultures

In 1907, Henry Van Peters Wilson discovered that siliceous sponge cells reaggregated, even after being dissociated. These cells could self-organize and differentiate into fully formed sponges (Wilson, 1907). Since then, multiple research teams have shown that various types of dissociated cells can be reaggregated in a similar manner. For instance, Holtfreter (1948) demonstrated cellular reaggregation in early embryonic amphibian cells (Holtfreter, 1943). Similarly, Weiss and Taylor (1960) performed dissociation-reaggregation experiments with cells from multiple organ sources obtained from chicken embryos (Weiss and Taylor, 1960). In 1964, Malcolm Steinberg proposed the differential adhesion hypothesis, suggesting that cell sorting and rearrangement are governed by thermodynamic principles driven by variations in surface adhesion (Steinberg and Locke, 1964).

The 1980s represented a major milestone in organoid research, with studies focusing on cell-matrix interactions in organoid development (Li et al., 1987) and the isolation of PSCs from mouse embryos in 1981 (Evans, 1981; Martin, 1981). In 1987, scientists started to improve cell culture conditions by simulating the *in vivo* microenvironment. Breast epithelial cells were demonstrated to form 3D ducts and lumens when cultured on an extracellular matrix (ECM) extract derived from Engelbreth-Holm-Swarm mouse sarcoma, enabling them to synthesize and secrete milk proteins, a capability not observed in traditional two-dimensional (2D) cultures (Li et al., 1987).

It was in 1998 when scientists successfully isolated and cultured embryonic stem cells from human blastocysts for the first time (Thomson et al., 1998). In 2006 and 2007, a breakthrough occurred when Takahashi and Yamanaka were able to develop iPSCs through the reprogramming of mouse (Takahashi and Yamanaka, 2006) and human fibroblasts (Takahashi et al., 2007). The isolation of stem cells greatly propelled organoid research, showing superior effectiveness and providing deeper insights for disease modeling.

The shift from 2D to 3D organoid cultures happened in 2008 when polarized cerebral cortex tissue was generated for the first time by Eiraku et al. from ESCs using serum‐free embryoid bodies with quick reaggregation (Eiraku et al., 2008). In 2009, intestinal ASCs were demonstrated to self-organize and differentiate to crypt-villus structures in the absence of a mesenchymal niche and they were used for the first time to generate 3D intestinal organoids in Matrigel® (Sato et al., 2009). This marked the first successful establishment of 3D organoid cultures derived from a single ASC, paving the way for disease modeling and precision medicine.

In 2013, Lancaster et al. generated the first brain organoids from human iPSCs (hiPSCs) upon growth in Matrigel® and with agitation, showing different brain regions (Lancaster et al., 2013). Since then, different studies have further refined organoid protocols to model specific brain areas, such as midbrain (Jo et al., 2016), hippocampus (Sakaguchi et al., 2015), and cerebellum (Muguruma et al., 2015), with innovations like the 3D-printing technology, which consists of a miniaturized spinning bioreactor, that has allowed the generation of forebrain organoids in a cost-effective manner (Qian et al., 2016).

## 3 Organoid cell sources

**FIGURE 1:**
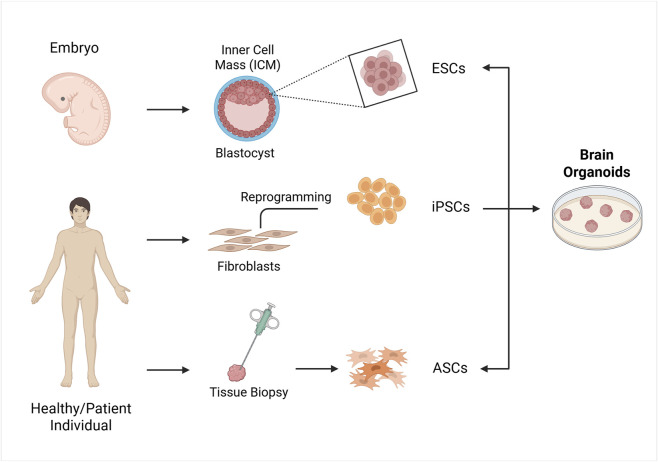
Strategies for the formation of organoids *in vitro*. The cell sources for establishing organoids include embryonic stem cells (ESCs), induced pluripotent stem cells (iPSCs) and adult stem cells (ASCs). ESCs are obtained from the blastocyst’s inner cell mass, iPSCs are generated by reprogramming somatic cells like fibroblast with transcription factors, and ASCs are obtained from organ biopsy samples from healthy or diseased tissues. These cells are then used to form organoids by incubating them with various signalling factors. Created in BioRender. Gomez Pinedo, U. (2025) https://BioRender.com/zcgbm6l.

The characteristics of an organoid, including its variability, heterogeneity, and functionality, depend significantly on the starting cell type, which can be either ASCs or PSCs, including both ESCs and iPSCs (Figure 1). PSC-derived organoids are generated by providing signaling cues that mimic *in vivo* development, and they are primarily used to study organogenesis and developmental events, as they resemble fetal-stage tissues, while ASCs-derived organoids reflect the self-renewal and differentiation capacity of somatic stem cells in tissue homeostasis (Watanabe et al., 2017).

## 4 Adult stem cells

ASC-based organoids are generated from biopsy samples obtained from either healthy or diseased tissues. The samples are dissociated into epithelia containing stem cells. These organoids closely simulate the original tissue’s dynamic stem cell behavior and are valuable models for studying monogenic diseases (Huch et al., 2015) and cancer (Matano et al., 2015) while also allowing molecular analysis and gene correction studies (e.g., using CRISPR/Cas9) (Schwank et al., 2013).

Robust protocols have been developed for the long-term cultivation, expansion, and cryopreservation of various ASC-derived organoid types. These protocols involve fewer steps, typically require less time overall and produce more mature structures that closely resemble the anatomy and function of adult tissue (Watanabe et al., 2017). This makes them highly suitable for regenerative medicine and disease modeling. ASCs are obtained either as isolated cells or from dissected tissue fragments and they are often used to generate organoids that maintain and repair their tissue of origin. For example, pancreas (Huch et al., 2013) and liver (Barker et al., 2010) organoids are used to model regeneration processes following injury. Besides, ASC-derived organoids can be generated from patient samples to assess responses to specific treatments, as seen in the colorectal cancer organoid biobank (Van de Wetering et al., 2015).

However, ASC-derived organoids have limitations. They have a restricted potency compared to PSCs (multipotency), as they are already predisposed to organ-specific differentiation and cannot generate multiple cell lineages, lacking the necessary tissue-tissue interactions to promote organ-level complexity. Moreover, as they require tissue samples containing stem cells, they have limited accessibility, and successful culture requires prior knowledge of tissue-specific conditions. While ASCs provide crucial insights into disease pathology, they are less suited for uncovering early disease mechanisms, as they primarily reflect tissue maintenance and regeneration in adulthood (Watanabe et al., 2017).

## 5 Pluripotent stem cells

PSCs have an unlimited capacity to self-renew and differentiate into all cell types of the body, making them a powerful tool for organoid generation (Chang et al., 2020). PSCs can be obtained with less invasive procedures, unlike ASCs. This accessibility along with their ability to generate diverse cell types, makes PSC-derived organoids ideal for modeling complex diseases such as NDDs. Due to their pluripotency, PSCs have high proliferation potential and possess the ability to differentiate into the three primary germ layers -ectoderm, mesoderm, and endoderm-including mesenchymal (mesoderm), epithelial (endoderm/ectoderm), and endothelial (mesoderm) (Watanabe et al., 2017). This makes them particularly suited for studying organogenesis in terms of not only cell differentiation, but also spatial patterning and morphogenesis (Clevers, 2016).

ESCs are derived from the inner cell mass of the blastocyst and have the potential to differentiate into all cell types (De Wert and Mummery, 2003). ESCs-derived organoids are very effective models for investigating genetic disorders and infectious diseases, especially in organs with very limited regeneration power like the brain (Ahammed and Kalangi, 2024). Nevertheless, their use raises strong ethical concerns due to the requirement of using human embryos (De Wert and Mummery, 2003).

IPSCs can be derived from various somatic cells, including keratinocytes, dental pulp stem cells and mesenchymal stem cells (MSCs). However, fibroblasts are the most used source for iPSCs generation, as they are generally easy to obtain and handle. Therefore, iPSCs are more accessible and ethically favorable compared to ESCs, as they are derived from adult tissues and do not require the use of human embryos. Additionally, organoids derived from ESCs exhibit more advanced maturation compared to those derived from iPSCs (Yang et al., 2023).

Initially, reprogramming somatic cells into iPSCs involved four transcription factors, OCT4, SOX2, KLF4 and c-MYC, also known as the Yamanaka factors. Since then, different cocktails have been used to successfully reprogram iPSCs (Ali et al., 2023). These factors can be delivered using methods like viral vectors, liposomes, or transposons (Pazzin et al., 2024). Integrating methods like retroviral and lentiviral delivery insert foreign genetic material into the cell’s genome, leaving an undesirable footprint that can cause mutations and safety risks, making them less suitable for clinical applications. To address this, non-integrating reprogramming methods, such as episomal plasmids, have been developed. These plasmids introduce reprogramming factors without integrating into the genome and are naturally lost over time, ensuring no permanent genetic alterations (Ali et al., 2023).

Since iPSCs can be derived from a patient’s own cells, they maintain the genetic and phenotypic traits of the donor tissue, making them an invaluable tool for personalized medicine, disease modeling, and patient-specific therapies (Halevy and Urbach, 2014). They better reflect disease-relevant phenotypic features, enabling more reliable disease modeling, allowing researchers to create patient and disease-specific cell lines, and avoiding immune rejection after transplantation. As iPSCs can differentiate into neural precursors, they subsequently generate diverse neuronal and glial cell types (Inoue et al., 2014). Numerous studies have already successfully reprogrammed iPSCs from fibroblasts obtained from individuals with various neurological disorders, including amyotrophic lateral sclerosis (ALS) (Dimos et al., 2008), Parkinson’s disease (PD) (Soldner et al., 2009) and AD (Choi et al., 2014). Furthermore, iPSC-derived organoids have been used for drug development and cell-based therapies by providing a renewable source of patient-matched cells (Dimos et al., 2008).

However, generating iPSC-based organoids remains complex, time-intensive, and expensive, requiring multiple reprogramming factors. Moreover, the resulting organoids are often immature and less functional, they have a limited lifespan and capacity to proliferate, frequently requiring additional culturing steps to achieve full maturation, and they lack interactions with other developing cell types (Yang et al., 2023). The use of oncogenes for reprogramming can also lead to *de novo* mutations (Halevy and Urbach, 2014). This instability increases tumorigenic risk, which presents a significant challenge for clinical applications, which may influence disease phenotype, posing a significant challenge for clinical application (Gutierrez-Aranda et al., 2010). To address these limitations, multiple biobanks have been established, supported by detailed patient clinical records. Additionally, utilizing established iPSC lines can help mitigate genetic variability (Evangelisti et al., 2024). These lines can be further modified using genome-editing technologies such as zinc finger nucleases, TALENs, and CRISPR/Cas to study specific genetic variants (Hotta and Yamanaka, 2015; Soldner and Jaenisch, 2018). Moreover, iPSCs fail to preserve the patient’s age, which hinders the study of aging-related diseases such as NDDs. This highlights the need to implement additional protocols to induce aging (Jothi and Kulka, 2024).

The wide variety of brain organoid cell sources can make it difficult to choose the most suitable one, as no single protocol fits all purposes. Researchers should select an organoid assay based on the complexity needed for their specific biological question and interpret results while considering the assay’s limitations.

## 6 From 2D TO 3D culture systems

Despite their advantages, like rapid growth, 2D culture systems, which are cultured on flat inert surfaces, face significant limitations (Saraswathibhatla et al., 2023), including the loss of normal polarity and shape and cell–cell and cell–extracellular interactions. This leads to cells that fail to accurately replicate the functions, development and behaviors observed in tissues or organs, compromising their biological relevance (Yang et al., 2023).

To overcome these limitations, more complex *in vitro* 3D culture systems were developed, replicating the *in vivo* physicochemical microenvironment (Drost and Clevers, 2018). These models maintain genetic stability and cellular structure while closely resembling the original cell types (Clevers, 2016; Drost and Clevers, 2018). They can be successfully cryopreserved and stored in living organoid biobanks and can be genetically characterized and used for drug screening and personalized medicine (Drost and Clevers, 2018). The ECM of the 3D culture enables inter-cellular signal transduction and physiological cues, simulating a more natural cell environment and providing a more reliable model system (Saraswathibhatla et al., 2023).

However, not all 3D neural culture systems qualify as brain organoids, as described by D’Antoni et al. in their review. Neurospheres, for instance, are 3D clusters of neural progenitor cells (NPCs) derived from central nervous system (CNS) primary tissue or iPSCs, but they lack the organization and complexity of true brain organoids. They grow in serum-free medium with FGF-2 and epidermal growth factor (EGF), without needing an adherent surface for expansion. Neurospheres are valuable for studying NPC behavior and neurogenic tissue in a simpler manner and are used in transplantation research (D’Antoni et al., 2023).

Thus, 3D brain organoids offer a more comprehensive model for investigating brain development and disease due to their structural complexity and capacity for long-term culture. They are generated from stem cells that self-organize through cell-sorting and form a heterogeneous population of cells that closely mimic the composition of the developing brain (“mini-brains”). As these structures evolve, they become more complex, leading to the formation of cortical progenitors. The most prominent structures emerging within the brain organoid tissue are ventricular-like zones and the organization of cortical layers (D’Antoni et al., 2023).

## 7 Generation of brain organoids

**FIGURE 2:**
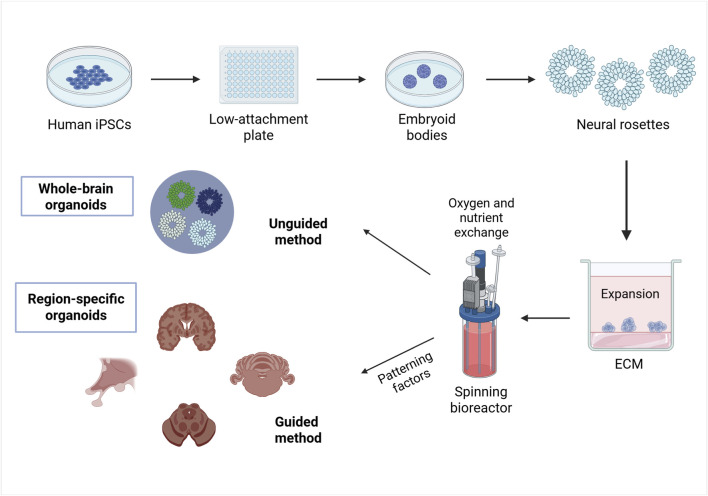
An example of a method for brain organoid specification. The method for generating brain organoids can be summarized as follows. Phase 1: creating PSCs, such as ESCs or iPSCs, into three-dimensional aggregates called embryoid bodies; Phase 2: inducing differentiation by applying environmental factors that stimulate formation of neuroepithelial buds o neural rosettes; Phase 3: maturation of differentiated brain tissue to exhibit the functionality of the nervous system by embedding the neural rosettes in an ECM and lacing the developing organoids in a spinning bioreactor. Ultimately, whole-brain organoids will be formed when using an unguided differentiation and region-specific organoids will be generated when using a guided differentiation. Created in BioRender. Gomez Pinedo, U. (2025) https://BioRender.com/pdai8s3.

The creation of PSC-derived 3D brain organoids could be summarized in three different steps (Figure 2) (Kwak et al., 2024). First, iPSCs or ESCs are aggregated to form embryoid bodies (EBs) by seeding them onto low-attachment plates for approximately 1 week. This process promotes free-floating 3D cell clusters in medium supplemented with low concentrations of basic fibroblast growth factor (bFGF) and a ROCK inhibitor (Huch et al., 2013; Kwak et al., 2024).

In the second phase of brain organoid generation, the EBs are transferred into neural induction medium, such as Dulbecco’s modified Eagle’s medium/Ham’s F12 (Kwak et al., 2024; Gómez-Álvarez et al., 2023), which provides essential nutrients and maintains a stable environment (Gómez-Álvarez et al., 2023). Antibiotics like penicillin/streptomycin are added to prevent bacterial contamination (Soldner et al., 2009; Gómez-Álvarez et al., 2023), while serum components, such as those present in fetal bovine serum, supply biomolecules necessary for cell growth and survival. Additionally, a variety of soluble factors are incorporated to promote cell signaling, differentiation, growth, viability, and function (Gómez-Álvarez et al., 2023).

B27, 2-mercaptoethanol and insulin are supplemented to encourage the differentiation into neuroectodermal lineages (Kwak et al., 2024; Lancaster and Knoblich, 2014a). Differentiation may be either guided using patterning factors to maximize the features of a specific brain region, or unguided if stem cells are allowed to self-organize into brain organoids containing multiple brain areas. Dorsal forebrain (pallium, cerebral cortex) organoids are induced by inhibiting wingless-type MMTV integration site family (WNT) signaling pathways, while ventral forebrain organoids are fabricated by inhibiting WNT signaling and activating sonic hedgehog (SHH) signaling pathways, which regulate the specification of dorsal and ventral brain regions. Hypothalamus organoids are induced by activating WNT and SHH signaling, along with the addition of ciliary neurotrophic factors and FGF2, which is crucial for cell proliferation and survival, and ciliary neurotrophic factors. Midbrain organoids are formed by modulating WNT and SHH signaling while supplementing the culture with FGF8, and cerebellar organoids develop through the introduction of FGF8 and WNT1, with FGF19 promoting further maturation (Kwak et al., 2024). Therefore, the precise selection and combination of the culture components must be tailored according to the tissue type and developmental stage to ensure optimal organoid formation and functionality (Lancaster and Knoblich, 2014a).

After differentiation, neuroepithelial buds or neural rosettes are embedded in ECM droplets, an ECM that provides support for cell differentiation and tissue organization. Another alternative for the development of organoids are hydrogels, bioprinting, suspension cultures and flat organoids. Growing organoids can be placed in a spinning bioreactor, where continuous agitation promotes growth, by enhancing oxygen and nutrient exchange, which ultimately supports the development of diverse brain regions resembling cerebral cortex (Kwak et al., 2024; Lancaster and Knoblich, 2014a). Finally, brain organoids mature over time replicating neural functionality (Kwak et al., 2024).

On the other hand, the transition from ASCs to organoids begins with enzymatic dissociation and purification of stem cells from the source tissue, followed by their cultivation in a 3D ECM or a different culture environment. In the second phase, a specialized culture medium containing specific growth factors such as Noggin, EGF, Wnt3a, or R-spondin is added to stimulate cell proliferation and differentiation. Finally, under these controlled conditions, ASCs self-organize and form 3D structures that recapitulate the characteristics of the original organ (Günther et al., 2022).

## 8 Unguided vs. guided differentiation in brain organoid generation

As commented before, brain organoid generation has been generally categorized into two groups based on patterning approaches, the unguided differentiation and the guided differentiation.

Unguided differentiation, which does not employ region-specific patterning factors but exploits the spontaneous intrinsic signaling of human PSCs, generates “whole-brain organoids” containing cells of the three primary brain vesicles: midbrain, hindbrain and forebrain (D’Antoni et al., 2023; Qian et al., 2019). These organoids predominantly generate neuro-ectodermal tissue, but they also produce non-ectodermal cell types, such as microglia (D’Antoni et al., 2023). However, the stochastic nature of the unguided differentiation often leads to inconsistent cell proportions, disorganized spatial distribution, altered initial conditions, and non-physiological cell interactions, resulting in higher variability and more complex reproducibility, which complicate experimental outcomes (Qian et al., 2019).

In contrast, guided differentiation involves the use of brain-specific patterning factors that stimulate developmental signaling pathways at precise stages of differentiation, allowing for the generation of brain organoids with specific regional identities (D’Antoni et al., 2023; Qian et al., 2019). This approach reflects better the specific domains of the nervous system at the anatomical, cellular and molecular level with higher reproducibility and reliability. However, because interactions between different brain regions are crucial for understanding neurodegenerative and psychiatric disorders, guided protocols have also been adapted to generate multiple brain-region-specific organoids that can be fused into assembloids after differentiation (Qian et al., 2019). These fused structures facilitate the study of complex cellular interactions and neurodevelopmental processes across different brain areas.

Ultimately, the choice of protocol, whether whole-brain organoids, region-specific brain organoids, or assembloids—depends on the specific scientific questions being investigated and the intended application in developmental biology, disease modeling, or therapeutic screening (Jang et al., 2022).

## 9 Cell culture strategies

### 9.1 Extracellular matrix supports

To successfully culture brain organoids, a supportive environment is required, typically involving solid ECMs that promote cell adhesion, growth, differentiation and migration. Matrigel®, that mainly consists of different kinds of laminins, but also of other proteins and factors in unknown quantities, is the most common matrix used for 3D organoid development. In some cases, type I collagen matrices have also been employed to create intestinal and mammary gland organoids. The key advantage of these natural matrices is their complex mixture of ECM components and growth factors, which promotes efficient cell expansion, spontaneous differentiation, and self-organization. However, this complexity, combined with poorly defined composition, heterogeneous nature, and batch-to batch variability, makes it harder to control the culture environment and reduces reproducibility. They also present potential risks such as introducing viral or xenogeneic contaminants that may trigger immune responses, disrupt organoid behavior, and limit the ability to induce organoid morphogenesis (Rossi et al., 2018; Aisenbrey and Murphy, 2020).

### 9.2 Chemically defined hydrogels

To overcome the previous challenges, chemically defined hydrogels have been developed as an alternative for supporting organoid cultures. Hydrogels are cross-linked hydrophilic polymer networks with a high-water content, mimicking the physicochemical properties of native tissues (Gómez-Álvarez et al., 2023). They allow for precise control of the culture environment’s biochemistry and mechanics. Notably, 3D screening techniques enable the synthesis and testing of hydrogels with varying stiffness, degradability, and bioactivity to assess their impact on stem cell fate (Ranga et al., 2014). The aim is to design a microenvironment that provides a closer approximation to the natural brain ECM to promote the development of more advanced and mature brain organoids.

Recent defined matrices have been derived from naturally occurring materials, such as proteins like fibrin (Broguiere et al., 2018), or polysaccharides like alginate (Capeling et al., 2019), chitosan (Zakhem et al., 2012) and agarose (Wylie et al., 2011). Additionally, decellularized ECM hydrogels obtained from whole organ decellularization have also been explored (Giobbe et al., 2019). Synthetic hydrogels have also been developed using materials like polylactic glycolic acid (PLGA), polyethylene glycol (PEG), polycaprolactone (PCL), and RADA16 (marketed as PuraMatrix). PLGA hydrogels, known for their excellent biocompatibility and biodegradability, have been used successfully for growing intestinal and liver organoids (Huch et al., 2015). PEG hydrogels, valued for their high-water content and customizable crosslinking, enhance nutrient and oxygen diffusion, particularly in intestinal organoid cultures (Wilson et al., 2021). PCL hydrogels, with their slow degradation, are well-suited for the long-term culture of neural organoids (Green and Abidian, 2015). Lastly, RADA16-based hydrogels support the formation of complex 3D neural structures in brain organoids (KarbalaeiMahdi et al., 2017). Both natural and synthetic biomaterials can be combined to create hybrid hydrogels that leverage the bioactivity of natural materials and the tunable properties of synthetic ones, leading to better organoid growth, stability, viability, and functionality (Zhang and Khademhosseini, 2017). An optimal organoid matrix should exhibit stress-relaxing behavior and dynamic biochemical and biophysical characteristics to accommodate structural changes during culture (Gjorevski et al., 2016). Hydrogels, particularly those that are crosslinked, mimic the viscoelasticity and dynamics of native ECMs, enabling relaxation under tissue-induced stresses while maintaining material stability (McKinnon et al., 2014).

### 9.3 Suspension cultures

Another strategy involves culturing 3D cell aggregates in suspension, which has been used for cerebral organoids (Eiraku et al., 2008). Although suspension cultures lack a solid scaffold, low concentrations of ECM are sometimes added to facilitate the formation of polarized epithelial structures (Eiraku et al., 2011). Additionally, 3D culture systems for organoids can utilize other strategies like the hanging drop method or the rotational culture method. The hanging-drop approach uses gravity and surface tension to suspend cell-medium droplets from a plate, promoting cell-cell interactions and aggregation, while rotational culture or bioreactors prevent cells from settling, enhancing nutrient and oxygen absorption through constant stirring or rotation, and has been used to the generation of brain organoids (Yang et al., 2023).

### 9.4 Emerging technologies

One major breakthrough in protocol standardization is bioprinting. This is an automated layer-by-layer deposition technique using cells and biomaterials to create 3D constructs. This technology enables the generation of organized structures, improving reproducibility (D’Antoni et al., 2023). Studies have successfully bioprinted stem cells, maintaining their multilineage potential (Reid et al., 2016; Nguyen et al., 2017; Koch et al., 2018) or pre-differentiating them before printing (Ma et al., 2016; Joung et al., 2018; Yu et al., 2019). However, there are significant challenges to overcome like developing bio-inks that support neuronal survival, differentiation, and maturation while mimicking the ECM. In the context of 3D bioprinting, hydrogels not only provide biocompatibility and structural support, but also enable neural network formation, neurite extension, and axon propagation. However, further refinements are needed for functional myelination and reproducibility (D’Antoni et al., 2023).

A different method for growing organoids is an air–liquid interface (ALI). In this approach, cells are formed into a pellet and cultured on a thin, microporous membrane, with culture medium provided only on the membrane’s basal side and the top of the mixture exposed to air (Ootani et al., 2009). Due to direct oxygen exposure, ALI cultures provide a higher oxygen supply compared to submerged culture methods, improving maturation and viability throughout the tissue during culture for up to 1 year. They enhance neuronal and astroglial survival and morphology, exhibiting extensive axon outgrowth making them suitable for neurological disease modeling (Giandomenico et al., 2019). Furthermore, microfluidic systems have improved organoid viability and differentiation significantly. They are designed to precisely control the biophysical and biochemical environment for cell growth. It simulates cellular and microenvironmental conditions, as well as interactions between tissues and multiple organs. Various organ-on-a-chip models have been developed to replicate specific organs *in vitro*, providing platforms for disease modeling and studying organ function. For example, Cho et al. developed a polydimethylsiloxane-based brain organoids-on-a-chip system that enhances oxygen supply, facilitates nutrient exchange, and reduces cell death, enabling the formation of mature brain organoids, moving closer to resembling native tissue and allowing human brain development (Cho et al., 2021).

## 10 Assembloids

Assembloids, considered the next-generation of organoids, are created by coculturing multiple cell types or combining organoids derived from different tissues (Yang et al., 2023). Small organoids with smooth, translucent edges, and strong integrity are selected for fusion. They are placed in close proximity within the same optimized culture media to facilitate adhesion and gradual integration. To prevent cell death and promote the formation of synaptic connections, physical and environmental conditions are regulated over time (Nityanandam et al., 2025).

Unlike single-cell-type organoids, assembloids reflect better interactions between subregions or cell lineages, facilitating the study of brain development processes such as long-distance projections and interneuron migration (Yang et al., 2023). Since interactions between brain regions and systems are critical in neurodegenerative and psychiatric diseases, establishing connections between brain organoids and other organ systems could significantly advance therapeutic target identification.

In the last couple of years, several studies have fused cerebral organoids from different brain regions, for example, Andersen et al. recently developed cortico-motor assembloids by combining cortical, hindbrain/cervical spinal cord, and skeletal muscle organoids, effectively modeling the cortico-motor pathway and the muscle contraction control (Andersen et al., 2020). Similarly, Ogawa et al. created a glioma model by integrating brain organoids with glioblastoma cells, offering insights into tumorigenesis and metastasis (Ogawa et al., 2018). Additionally, cortex-ganglionic cortex assembloids reproduced brain network formation and exhibited epileptiform-like activity, contributing to research on Rett syndrome (Samarasinghe et al., 2021).

Assembloids research has also been extended to the peripheral nervous system. iPSCs have been differentiated into spinal cord neurons and skeletal muscle cells that self-organize into neuromuscular organoids (Faustino Martins et al., 2020) and intestinal organoids cocultured with neural crest cells have modeled enteric nervous system development and intestinal motility disorders (Workman et al., 2017).

Understanding body-wide interactions is essential for studying neurological diseases, as key therapeutic targets may exist outside the brain (Yang et al., 2023). For example, liver disfunction has been linked to AD and PD (Bassendine et al., 2020; Ciulla et al., 2019). Organ-on-a-chip systems, which connect organoids through microfluidic channels and mimic microenvironment conditions (D’Antoni et al., 2023; Yang et al., 2023), enhance our understanding of neurological and psychiatric disease mechanisms by modeling inter-tissue and multi-organ interactions (Yang et al., 2023).

## 11 Introducing microglia and vascularitation in brain organoids

Unlike neurons, which originate from the neuroectoderm, microglia derive from embryonic yolk sac progenitors and migrate to the brain, where they self-renew with the help of cytokines (IL-34, CSF-1) and transcription factors (PU.1, IRF8). As the brain’s primary immune cells, microglia play a crucial role in neuroinflammation, synaptic regulation, and neural circuit formation. However, since brain organoids lack native microglia, researchers have introduced microglial precursors to create microglia-containing assembloids, useful for modeling NDDs (D’Antoni et al., 2023).

Multiple strategies have integrated microglia into brain organoids, advancing research on brain-immune interactions. These studies range from co-culture systems to genetic engineering techniques. In 2017, Abud et al. introduced microglia into iPSC-derived organoids via a co-culture system, showing mature microglial behavior validated by cytokine secretion and synaptic pruning assays (Abud et al., 2017). In 2018, Ormel et al. generated microglia-containing organoids by reducing heparin levels and delaying ECM embedment, resulting in microglia with realistic morphology and immune responses (Ormel et al., 2018). In 2019, Song et al. introduced iPSC-derived microglia into cerebral organoids, demonstrating immune responses and their ability to stimulate cell proliferation and reduce oxidative stress (Song et al., 2019). In 2021, Ao et al. developed a tubular organoid-on-a-chip system with isogenic microglia, reducing hypoxia and modelling neuro-immune interactions by supporting microglial activation after exposure to an opioid receptor agonist (Ao et al., 2021). In 2022, Cakir et al. improved microglia integration by inducing PU.1 expression, enhancing reproducibility and consistency in organoid models (Cakir et al., 2022). In the future, advanced protocols are expected to incorporate region-specific glia, aiming to enhance the molecular and functional characteristics of neurons in brain organoids. These findings collectively demonstrate the feasibility of integrating microglia into brain organoids, although challenges in consistency and functionality remain (D’Antoni et al., 2023).

Another challenge that arises is cell death in the internal regions due to lack of vascularization that alters oxygen and nutrient delivery, metabolite elimination, and cell signaling. This restricts the organoids lifespan, making it difficult to use organoids for long-term studies (Lancaster and Knoblich, 2014b). To overcome this, new strategies have been developed.

Coculturing brain organoids in a ECM with endothelial cells has promoted vascularization by using FGF2, CHIR99012, BMP4, and VEGF to promote proper endothelial differentiation (Lancaster and Knoblich, 2012; Pham et al., 2018). Other studies have been based on the co-culture of human umbilical vein endothelial cells (HUVECs) with PSCs before neural induction, producing vascularized brain organoids (HUVEC-BOs) with enhanced neurogenesis and maturation (Shi et al., 2020). An alternative approach is generating brain-specific vascular organoids. In a guided protocol, human ESCs were differentiated into human blood vessel organoids, which were then fused with cerebral organoids stimulating vascularization (Sun et al., 2022). Different studies have developed BBB organoids by combining glial, vascular, and neuronal cells in co-culture systems, or by producing BBB assembloids through the fusion of brain and blood vessel organoids. This type of model could be valuable for studying vascular dysfunction and BBB disruption associated with some NDDs, such as AD, PD and MS, as it closely mimics the interactions between neural and vascular components (Summers et al., 2024). Bergmann et al. created BBB organoids by coculturing brain endothelial cells, astrocytes and pericytes under low conditions, enabling studies on drug permeability and neurodegenerative therapies (Bergmann et al., 2018). Microfluidic devices have also been proven to recapitulate the BBB complexity, allowing the study of the interaction of compounds with endothelial cells, pericytes and astrocytes, and the transition across the BBB(101).

Several additional approaches have been proposed to enhance vascularization in organoids. As commented before, one strategy involves utilizing microfluidics devices or organ-on-a-chip devices with periodic flow that improve nutrient and oxygen exchange in brain organoids while reducing cell death (Cho et al., 2021). Pericytes and endothelial cells derived from human PSCs spontaneously form vascular networks that physically integrate with cerebral organoids, creating fully connected neurovascular organoids on a chip (Castiglione et al., 2022). Another approach involves genetically engineered cortical organoids to express human ETS variant transcription factor 2 (hETV2) to spontaneously form a vascular-like network *in vitro* which dramatically reduces markers of cell death and hypoxia without transplantation (Cakir et al., 2022).

Current brain organoids do not have cerebrospinal fluid (CSF). Co-culturing vascularized brain organoids and choroid plexus organoids has allowed the production of a more complete vasculature in cerebral organoids (Pellegrini et al., 2020).

Other progresses that have been made in this area are spinning bioreactors that enhance nutrient exchange and enable growth up to a few millimeters in size (Lancaster and Knoblich, 2014a), ALI cultures (Cho et al., 2021), and slicing or cutting methods, to culture organoids as small pieces, reducing hypoxia and necrosis (Choe et al., 2021). Silk scaffolds (Sozzi et al., 2022), PCL scaffolds (Rothenbücher et al., 2021) and carbon fibers (Tejchman et al., 2020) have also been used as innovative frameworks to reduce necrosis and promote neuronal survival in organoids by improving oxygen flow and waste removal. Lastly, the transplantation of organoids in animals has also resulted in vascularization (Mansour et al., 2018; Revah et al., 2022).

The integration of microglia and vascularization in brain organoids represents a major step toward more physiologically relevant models. Advances in co-culture techniques, microfluidics, and genetic engineering are progressively overcoming current limitations, paving the way for improved disease modeling and therapeutic testing.

## 12 Myelinoids

Myelination is a process where oligodendrocytes wrap axons in the CNS with myelin, a fatty substance that speeds up the transmission of nerve impulses (López-Muguruza and Matute, 2023). This process is crucial for the proper functioning of the nervous system, and its disruption is associated with various NDDs, such as MS (Nasrabady et al., 2018). Despite significant advancements, many aspects of myelin formation and function remain poorly understood, partly because models fail to replicate accurately human physiology. Consequently, there has been a long-standing need for robust human cell-based models of myelination to study myelin formation and the pathological processes underlying human diseases.

Organoids are particularly well-suited for research as they mimic aspects of spatial complexity and cellular diversity found in living organisms. In 2018, Madhavan et al. developed “oligocortical spheroids” by differentiating human PSCs and adding compounds, such as T3, clemastine or ketoconazole. These spheroids matured into functional oligodendrocytes, demonstrating myelin compaction and response to promyelinating drugs. Additionally, patient-derived spheroids mimicked disease-related abnormalities, making them a valuable tool for studying disease mechanisms (Madhavan et al., 2018). In 2019, Kim et al. created forebrain organoids using human PSCs engineered with an OLIG2-GFP knockin. These organoids successfully modeled human oligodendrogenesis, demonstrating that oligodendrocytes can originate from both ventral and dorsal regions. This model provided a valuable platform for investigating cortical myelin defects and regional oligodendroglial differences (Kim et al., 2019a). In the same year, human iPSCs were used by Marton et al. to generate brain organoids containing oligodendrocytes, neurons and astrocytes. Oligodendrocytes matured and began myelinating nearby neurons (Marton et al., 2019).

In 2021, James et al. developed myelinating organoids (“myelinoids”) derived from human iPSCs with genetic mutations to study oligodendrogenesis, compact myelin formation and myelinated axon organization. Pharmacologic perturbations altered myelin formation by reducing neuronal synaptic vesicles (James et al., 2021).

In 2023, Feng et al. generated human iPSC-derived myelin spheroids to model Canavan disease, a demyelinating disorder. By treating them with N-acetyl-aspartate, they were able to mimic key pathological features of the disease, establishing this model as a tool to study myelin diseases (Feng et al., 2023).

Myelinoids have been demonstrated to provide a robust platform for studying oligodendrocyte development, mechanisms of myelination, and cell-cell interactions in the CNS, holding potential for advancing research into white matter disorders, such as MS, and for developing therapeutic interventions.

## 13 Organoid applications

**FIGURE 3:**
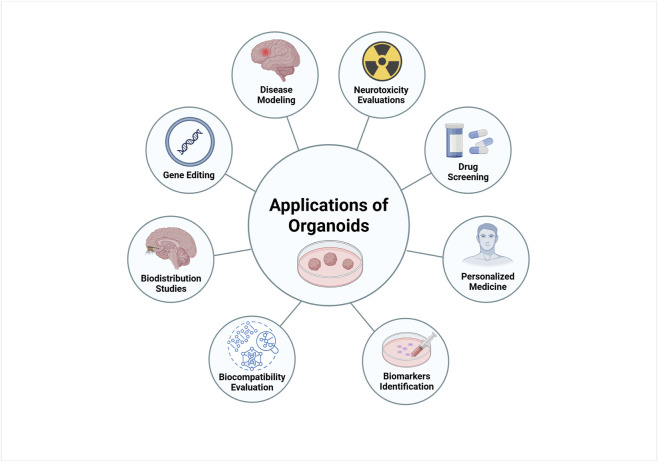
Biomedical applications of brain organoids. Organoids can be used as disease models to understand the mechanisms and physiopathology of human neurodegenerative diseases. Organoids are ideal models for drug screening and toxicity assays. Patient-derived organoids can be used to predict patient-specific responses to drugs and personalized treatment. Other biomedical applications of organoids include biomarker discovery, biodistribution study and regenerative medicine. Created in BioRender. Gomez Pinedo, U. (2025) https://BioRender.com/gkjv7i5.

Organoids are rapidly becoming a key tool in cell culture for a wide range of biomedical research. Their ability to represent diverse tissue types, sustain long-term growth, and recapitulate physiological 3D structures makes them an innovative technology with numerous biological and clinical applications. Importantly, organoids have found extensive use in areas such as disease modeling, precision medicine, toxicology testing, and regenerative medicine (Corrò et al., 2020). Next, we will describe their applications in disease modeling, biobanking, personalised medicine, neurotoxicity, identification and validation of biomarkers, drug screening, gene editing and biocompatibility (Figure 3).

### 13.1 Neurotoxicity evaluations

Neurotoxicity studies aim to elucidate the adverse effects and underlying mechanisms of toxic agents on the CNS, which is highly sensitive to external insults. Traditionally, these evaluations have relied on animal models; however, fundamental interspecies differences raise concerns about their translational relevance to human physiology. To overcome these limitations, human cell-based models such as organoids offer a cost-effective, ethical, and more physiologically relevant alternative (Cao, 2022).

3D brain organoids reproduce key features of human tissues, including cellular heterogeneity, structural complexity, and functional characteristics, with greater physiological and pathological traits. These properties make them a powerful platform for drug screening and toxicity testing, particularly in the context of developmental neurotoxicants (Chhibber et al., 2020). Disease-specific organoids derived from patient iPSCs further enhance the utility of this model, as they recapitulate clinical phenotypes observed in primary cancers, infectious diseases, and neurodevelopmental disorders. Despite these advantages, preclinical studies using organoids still face challenges, as many toxic effects only become apparent during clinical trials or post-market surveillance (Tang et al., 2022).

Organoids also facilitate toxicity assessments across multiple organ systems—including liver, heart, and kidneys—supporting the development of safer drugs through long-term screening approaches that more closely mimic human physiology (Tang et al., 2022). Beyond drug toxicity, organoids have been instrumental in modeling neurodegeneration induced by environmental agents. These studies have uncovered molecular and cellular mechanisms of toxicity and, with the aid of machine learning, have identified previously unrecognized neurotoxicants, demonstrating their potential for high-throughput screening (Monzel et al., 2020).

In AD research, cerebral organoids have been exposed to small-molecule inducers of amyloid-beta (Aβ) accumulation to investigate the role of environmental risk factors. These models exhibit hallmark AD phenotypes, such as synaptic loss and tau phosphorylation, enabling the study of underlying neurotoxic mechanisms (Pavoni et al., 2018). Similarly, midbrain organoids have been employed to model PD by treatment with toxins like rotenone and MPP+, which induce dopaminergic neuron degeneration. These organoids recapitulate key PD pathologies, including mitochondrial dysfunction and oxidative stress, supporting their use in evaluating environmental contributors to neurodegeneration and screening potential neuroprotective strategies (Kwak et al., 2020).

### 13.2 Drug screening

Despite the evident interpatient heterogeneity, most clinical drugs are not developed using molecular biomarkers, except for those targeting specific mutations in particular pathways. To personalize treatment, drug sensitivity assays using patient-derived organoids (PDOs) are progressively improving by providing a closer approximation to pathological characteristics of tumors. Therefore, PDOs should be used in drug screening to guide clinical treatment and improve patient prognosis. Traditionally, precision therapies have been based on mutational biomarkers. As a result, treatments targeting these markers do not always yield favorable patient responses. PDO models have been used in drug discovery to explore the cytotoxicity of therapeutic candidates and facilitate personalized cancer treatments (Zhou et al., 2021).

High-throughput screening (HTS) has been widely adopted in drug discovery, enabling the rapid evaluation of thousands of chemical compounds in PDO-based assays. This approach helps identify lead compounds and assess their therapeutic potential with higher efficiency. HTS applied to patient-derived organoids enhances the discovery of effective drugs by integrating molecular profiling data, ensuring the relevance of selected compounds to patient-specific tumor biology (Engle and Vincent, 2014).

In drug screening, a biobank of organoids derived from pancreatic ductal adenocarcinoma enabled the evaluation of 76 therapeutic agents, identifying the PRMT5 inhibitor (EZP015556) as effective in both MTAP-positive and MTAP-negative organoids, emphasizing the importance of personalized approaches in oncology. These findings underscore the value of PDOs as tools for disease modeling and advancing precision medicine (Tang et al., 2022). Similarly, Zhou et al. presented a comprehensive overview of various drug screening studies, highlighting key findings and methodologies (Zhou et al., 2021).

Microelectrode arrays (MEA) have also been integrated into organoid-based drug screening to assess electrophysiological responses to therapeutic compounds. Recent studies on brain organoids demonstrated that MEA platforms can monitor neuronal activity changes upon drug treatment, providing real-time insights into drug efficacy and toxicity in neurological disorders. Advances in 3D shell MEAs have significantly enhanced signal detection and stability, allowing precise electrophysiological assessments of drug responses in encapsulated brain organoids (Huang et al., 2022).

Furthermore, gastric cancer (GC) organoids have been used to evaluate drug sensitivity and resistance mechanisms. RNA sequencing and whole-exome sequencing (WES) have confirmed that GC organoids retain the genetic characteristics of the original tumors, making them reliable models for testing chemotherapeutic responses (Kim et al., 2015).

However, organoid-based drug screening is still evolving. Variability in culture conditions and batch-to-batch differences remain concerns, affecting reproducibility. While initial studies have demonstrated their potential in small-scale drug screening setups, the development of standardized protocols and automation will be essential to improve their reliability, enabling their broader application in toxicity assessments and therapeutic development (Liu et al., 2019; Wang et al., 2018).

### 13.3 Personalized medicine

PDOs exhibit rapid growth, stable differentiation, and the ability to capture interpatient and tumor heterogeneity, making them powerful tools for personalized medicine. They allow for drug sensitivity testing that identifies optimal therapies for individual patients, reducing adverse effects and improving outcomes. Moreover, PDOs serve as reliable platforms for preclinical drug screening and development of novel treatments (Tang et al., 2022).

The creation of organoid biobanks from various tumors—such as prostate (Beshiri et al., 2018), lung (Li et al., 2020), colorectal (Fujii et al., 2016), liver (Broutier et al., 2017), pancreatic (Driehuis et al., 2019), and gastric cancers (Yan HHN. et al., 2018) —has expanded their utility. These “living biobanks,” comprising cryopreserved and expandable organoids, enable in-depth pathological investigations and support clinical decision-making when integrated with drug screening and next-generation sequencing (Zhou et al., 2021).

A landmark application is in cystic fibrosis (CF), where rectal PDOs predicted better the individual responses to CFTR modulators, marking the first clinical use of organoids to guide therapy (Berkers et al., 2019). Similarly, in rectal cancer, PDOs mirrored patient responses to chemoradiotherapy, demonstrating their potential to forecast treatment outcomes (Ganesh et al., 2019; Park et al., 2021; Yao et al., 2020).

CRISPR-Cas9 gene-editing has further enhanced PDO applications by enabling the introduction of patient-specific mutations. This enables the development of models that capture key genetic features for studying disease mechanisms and testing targeted therapies (Li G. et al., 2022). For instance, colorectal cancer organoids have been edited to study tumor progression and drug resistance (Matano et al., 2015); pancreatic cancer models have revealed changes in stem cell niche dependence (Seino et al., 2018); and lung cancer organoids have been engineered to reflect mutational profiles for drug testing (Hu et al., 2021). In gastric cancer, CRISPR-edited PDOs preserved key mutations (e.g., TP53, TTN, CSMD1), with whole-exome sequencing confirming their genomic fidelity (Li G. et al., 2022). Likewise, renal cell carcinoma organoids with mutations in VHL, PBRM1, and AHNAK2 have been used to test targeted therapies (Li Z. et al., 2022).

Despite these advances, challenges persist in ensuring the long-term genetic stability and reproducibility of PDO biobanks. Enhancing cryopreservation protocols and automating processes will be essential. Furthermore, while gene editing has enabled the creation of personalized models, translating these advances into routine clinical applications requires further validation to ensure they capture the full complexity of patient-specific conditions.

### 13.4 Identification and validation of biomarkers

The ability of organoids to partially reproduce the tissue microenvironment positions them as valuable models for the identification and validation of disease biomarkers. These 3D models allow a more relevant analysis of cellular and molecular interactions, facilitating the detection of specific markers associated with various pathologies (Romero et al., 2019).

A prominent example is the work carried out by the Barcelonaβeta Brain Research Center, the research institute of the Pasqual Maragall Foundation, dedicated to the prevention of AD and the study of cognitive functions affected in healthy and pathological aging. At this center, brain organoids are being developed from stem cells which simulate the development of AD and explore in detail the factors that contribute to its onset and progression. This innovative approach aids in the identification of specific biomarkers for the disease, improving diagnostic accuracy and opening new avenues for the development of more effective therapies (Romero et al., 2019).

Additionally, the implementation of organoids in cancer research has proven efficacy in identifying biomarkers that predict treatment response and tumor progression. The ability of these models to reflect tumor heterogeneity and the specific cancer microenvironment allows a more significative assessment of the underlying molecular mechanisms (Romero et al., 2019).

The integration of biosensor-functionalized microfluidic platforms with organoids—commonly referred to as organoids-on-a-chip—has significantly enhanced their analytical capabilities. These systems enable real-time, non-invasive monitoring of microenvironmental parameters such as temperature, pH, and oxygen, as well as continuous detection of specific biomarkers, reducing the need for disruptive sampling. In hepatic organoids, biomarkers like albumin (indicative of liver function) and glutathione S-transferase α (GST-α, a marker of liver injury) can be tracked over time, facilitating the evaluation of drug-induced liver damage. Similarly, in cardiac organoids, the dynamic detection of creatine kinase MB (CK-MB), a biomarker of cardiac injury, supports the continuous assessment of cardiotoxic effects. By closely mimicking physiological conditions through continuous nutrient perfusion and waste removal, these platforms improve organoid viability and provide powerful tools for biomarker discovery and drug safety testing (Zhang et al., 2017).

Furthermore, genetic engineering tools like CRISPR-Cas9 have expanded the role of organoids in biomarker discovery by enabling the study of disease-associated mutations. For instance, researchers have utilized CRISPR-Cas9 to introduce mutations in genes such as TP53 within organoid models, leading to the development of tumor-like phenotypes. This approach allows for the assessment of tumor behavior and response to various treatments, thereby aiding in the identification of potential biomarkers for early diagnosis and therapeutic targeting (Drost et al., 2015).

These advances highlight the potential of organoids in identifying disease-specific biomarkers, improving diagnostic accuracy, and enhancing treatment efficacy for various conditions. While these applications are promising, challenges include ensuring reproducibility across different organoid models and standardizing protocols for biomarker validation. Additionally, the complexity of integrating biosensors, microfluidic systems, and genetic engineering approaches requires further optimization to ensure scalability and accessibility for widespread biomedical applications.

### 13.5 Evaluation of biocompatibility

In the field of regenerative medicine, organoids have emerged as a promising tool for assessing the biocompatibility of biomaterials intended for implants and prosthetics (Lancaster and Knoblich, 2014b).

Organoids are increasingly being used to evaluate the biological response to various biomaterials, ensuring these materials do not trigger adverse reactions such as inflammation or cellular toxicity. Recent studies have highlighted the potential of organoids in assessing the interactions between biomaterials and human tissues, which is essential for the safe and effective development of implants. Additionally, integrating organoids with tissue and organ chips has led to more realistic models for drug discovery, toxicity testing, diagnostics, therapeutics, and personalized medicine, effectively bridging the gap between *in vitro* experimentation and clinical applications (Sean et al., 2024).

For example, a study published in Nature Materials in 2020 used human intestinal organoids to assess the biocompatibility of medical-grade polymers. The results showed that certain polymers caused minimal inflammation and supported cellular growth, suggesting their suitability for biomedical applications (Schutgens and Clevers, 2020). This study highlights how organoids can be used to predict the behavior of materials in human tissues, reducing the need for animal testing. However, ensuring consistency in organoid responses remains a challenge. Future advancements in integrating organoid systems with microfluidic platforms may help standardize biocompatibility assays.

Furthermore, the integration of organoids into the development of biomaterials has opened up new possibilities for personalized medicine. By incorporating patient-specific organoids derived from stem cells, researchers are now able to assess how different materials interact with tissues in an individual-specific context (Sato et al., 2009). This approach is particularly valuable for personalized implant designs, where biomaterial selection can be tailored to each patient’s unique tissue environment.

In conclusion, organoids are a fundamental tool in regenerative medicine for evaluating the biocompatibility of biomaterials. Their integration into preclinical testing holds great promise for accelerating the development of safer, more effective implants and prosthetics, while offering innovative solutions to enhance regenerative and surgical treatments.

### 13.6 Biodistribution studies

Organoids are emerging as a promising tool for conducting biodistribution studies due to their ability to mimic more faithfully the architecture and functionality of human organs on a 3D scale. Unlike traditional models based on 2D cell monolayers, organoids exhibit a more complex and dynamic structure that better reflects cell-to-cell and cell-to-ECM interactions, enabling a closer simulation of *in vivo* conditions. This feature makes them more relevant models for compound distribution studies within human tissues, providing a more controlled and representative analysis compared to conventional methods (Berger et al., 2022; Nguyen et al., 2022).

Previous studies have successfully employed organoids from human organs such as the kidney and liver in multi-organ-on-a-chip models to investigate the biodistribution and therapeutic effects of mesenchymal stem cell-derived extracellular vesicles. This setup provides a more physiologically relevant context for assessing compound behaviour and therapeutic responses prior to clinical application (Berger et al., 2022; Nguyen et al., 2022).

These models allow for the analysis of how these substances distribute within brain tissue, which is key to determining their therapeutic potential in neurological disorders and other types of cancer. The use of brain organoids in this context opens new opportunities to evaluate treatment safety and efficacy before clinical trials, providing a more effective and representative tool for predicting therapeutic responses (Berger et al., 2022; Nguyen et al., 2022).

The integration of organoids with advanced technologies, such as microfluidic organ-on-a-chip system, has enabled high-throughput screening, significantly accelerating preclinical research. These microfluidic systems not only simulate the physiological and pathological conditions of organs but also allow for the integration of different cell types and the recreation of complex microenvironments, such as vasculature, making them a promising alternative to traditional animal models. This combination of organoids and microfluidics can expedite the identification of therapeutic compounds and improve the precision of biodistribution studies, which is essential for the development of personalized therapies and precision medicine (Berger et al., 2022; Nguyen et al., 2022).

In summary, organoids represent an advanced and robust platform for conducting biodistribution studies due to their ability to recapitulate better the *in vivo* biological conditions. Their use, combined with technologies such as “organ-on-a-chip” systems, not only offers an effective alternative to animal models but also enhances our understanding of the distribution and behavior of drugs in various tissue types, which could accelerate the discovery of new treatments and improve the effectiveness of therapies in personalized medicine (Berger et al., 2022; Nguyen et al., 2022).

### 13.7 Gene editing

CRISPR/Cas9 gene editing has become an essential tool in PDOs, enabling the creation of tumor transformation models, targeted therapy evaluations, and the correction of pathogenic mutations (Zhou et al., 2021). For instance, Kuo et al. created the first human genetic model of the commonly mutated tumor suppressor gene ARID1A in gastric cancer, providing insights into early transformation processes (Lo et al., 2021). Similarly, Visvader et al. used CRISPR/Cas9 to knock out breast cancer-related tumor suppressor genes and developed PDOs capable of long-term growth (Dekkers et al., 2020), while Meltzer et al. modeled aberrant Wnt signaling in Barrett’s epithelium transformation (Liu et al., 2018).

In addition to cancer, CRISPR/Cas9 has been widely applied in brain organoids to study neurodevelopmental disorders, as these models closely mimic early human brain development (Lancaster et al., 2013). Comparative studies between human, chimpanzee, and macaque-derived organoids have revealed significant differences in cell organization and gene expression, providing insights into human brain evolution (Pollen et al., 2019). Furthermore, the introduction of Neanderthal genes into human organoids has been explored to understand their impact on modern human cognition (Cohen, 2018).

In regenerative medicine, gene editing allows for the correction of mutations in patient-derived stem cells before generating transplantable tissues. For example, CRISPR/Cas9 repair of CFTR mutations in cystic fibrosis patient-derived intestinal organoids restored ion channel function, while retinal organoids were corrected for mutations linked to inherited blindness, demonstrating its potential for degenerative diseases (Yin et al., 2016; Nie and Hashino, 2017).

CRISPR/Cas9 is also used to introduce or correct disease-associated genetic variants, enabling functional studies. In hepatic organoids, metabolic liver diseases have been modeled, and in pancreatic organoids, genetic factors related to diabetes have been explored (Nie and Hashino, 2017). These models not only improve disease mechanism understanding but also provide platforms for high-throughput drug screening.

In NDDs like AD, the conversion of the APOE3 allele to APOE4 — the strongest genetic risk factor—in iPSC-derived brain models allowed direct comparisons of their effects on neurons and astrocytes, uncovering functional and transcriptomic differences relevant to disease progression (Lin et al., 2018). Additionally, the induction of PU.1 in cortical organoids has generated microglia-like cells, enabling the study of neuroinflammation and its role in amyloid plaque formation and tau phosphorylation (Cakir et al., 2022).

One major challenge in organoid transplantation is immune incompatibility. CRISPR/Cas9 has been employed to delete major histocompatibility complex (MHC) genes in liver organoids, reducing rejection risk post-transplant. Immune-evasive gene modifications, such as the expression of PD-L1, have also been explored to inhibit host immune responses. Furthermore, the creation of “universal donor” organoids through genetic modification of iPSCs to eliminate rejection markers has shown improved survival in liver and kidney organoid transplantation models, marking progress toward personalized regenerative medicine (Tsuchida et al., 2020).

### 13.8 Disease modeling

In the context of disease modeling, brain organoids have been extensively used to study conditions such as microcephaly (Lancaster et al., 2013), autism (Wang et al., 2017) and schizophrenia (Stachowiak et al., 2017). Additionally, they have provided valuable insights into NDDs like AD (Raja et al., 2016) and PD (Kim et al., 2019b; Marotta et al., 2020). The flexibility of brain organoids as models allows researchers to use patient-derived cells or introduce disease-related mutations to investigate pathological mechanisms. This approach has also been crucial in studying viral infections such as the Zika virus, where infected brain organoids have helped identify key morphological and genetic alterations associated with the disease (Qian et al., 2016; Dang et al., 2016).

Brain organoids derived from hPSCs, particularly patient-derived iPSCs, have been extensively studied for their potential to model neurodevelopmental disorders. They have proven particularly effective in recapitulating disease-related phenotypes in conditions where structural malformations are evident during early embryonic stages. The underlying mechanisms of these disorders are often attributed to altered regulation of progenitor cells, including premature differentiation, reduced proliferation, and cell cycle disruptions, all of which can be reliably analyzed using brain organoids (Qian et al., 2019).

In addition to genetic conditions, brain organoids have been used to model the effects of neurotropic pathogens on brain development. Through the use of genetic manipulation methods, such as viral vectors or electroporation, organoids also serve as accessible models for studying the function of specific proteins or pathways and for investigating the molecular mechanisms underlying infective diseases (Qian et al., 2019).

Modeling neurodevelopmental disorders that do not involve significant structural malformations remains a challenge. However, brain organoids have provided valuable insights into the cellular and molecular mechanisms involved in such disorders (Qian et al., 2019).

Although brain organoids have generated significant interest as models for NDDs, progress has been limited. Many of these conditions are late-onset and age-related, meaning that organoids mimicking embryonic brain development may not robustly replicate the relevant disease phenotypes. Nevertheless, human neuronal cultures and neurospheres derived from individuals with AD have successfully reproduced AD-like pathologies, including amyloid aggregation, hyperphosphorylation of tau protein, and endosomal abnormalities (Qian et al., 2019).

Midbrain organoids containing tyrosine hydroxylase-positive dopaminergic neurons, when combined with pharmacological treatments that induce neurodegeneration, could serve as models for PD and as a cellular source for replacement therapies (Qian et al., 2019).

#### 13.8.1 Alzheimer’s disease

AD is a progressive neurodegenerative disorder characterized by cognitive decline and is the leading cause of dementia. While most AD cases are sporadic, a small percentage (about 1%) are familial, driven by mutations in the PSEN1, PSEN2, and APP genes. The Amyloid Hypothesis has long been the dominant theory, suggesting that Aβ accumulation triggers neurodegeneration and cognitive decline (Selkoe and Hardy, 2016). Despite efforts targeting Aβ pathology, tau hyperphosphorylation and aggregation have gained increasing focus, as tau pathology may develop independently of Aβ and correlate more strongly with neurodegeneration (van der Kant et al., 2020). Genome-wide association studies and other research have highlighted the complexity of AD, with genetic risk factors, aging, cellular states, and cell-cell interactions all contributing to the disease (Mh and Lh, 2023). Key factors include the APOE gene E4 variant, which affects lipid metabolism, myelination, and neuroinflammation, and various cell types, including astrocytes, oligodendrocytes, microglia, and the brain vasculature, which play pivotal roles in AD pathology (Yamazaki et al., 2019). The Myelin Breakdown Hypothesis emphasizes the importance of myelin integrity, while neuroinflammation, triggered by myelin breakdown and microglial activation, contributes to cognitive decline (Depp et al., 2023). Vascular dysfunction, including BBB breakdown and cerebral amyloid angiopathy, further exacerbates the disease (De Strooper and Karran, 2016). Understanding the complex interactions between these cellular phenotypes is crucial for advancing therapeutic strategies for AD.

The groundbreaking development of iPSC technology has enabled *in vitro* disease modeling using patient-derived cellular models. Human iPSC-based disease models address the challenges associated with obtaining primary human tissues, such as brain tissue, and overcome species-specific differences commonly observed with animal models. While 2D cell culture models have dominated *in vitro* research, more intricate cellular structures are required to accurately replicate the multifaceted pathogenesis of AD. The advent of human organoid technology bridges the gap between 2D models and the complex 3D *in vivo* environment, offering a closer representation of disease processes (Cerneckis et al., 2023).

Recent advancements in brain organoid research have led to the development of various models to better understand the pathogenesis of AD and explore potential therapeutic strategies (Table 1). Among these, genetically modified brain organoids derived from human iPSCs and neural precursor cells have proven particularly valuable. By introducing mutations such as those in APP and PSEN1, researchers have successfully generated models that reproduce hallmark AD features, including beta-amyloid accumulation and tau pathology (Qian et al., 2019; Raja et al., 2016; Gonzalez et al., 2018).

**TABLE 1:** Summary of organoid models used in Alzheimer’s Disease research. The table summarizes general information regarding the cell source, induction method, support method, main findings obtained using each approach, potential applications, and corresponding references.

Disease: Alzheimer’s Disease					
Cell source	Induction method	Support method	Key findings	Applications	References
Human neural precursor cells	Generation of APP and PSEN1 mutations	ECM	Beta-amyloid and tau protein Treatment with β- and γ-secretase inhibitors	Models of neurodegenerative disorders Testing potential therapies targeting Aβ and tau	Qian et al. (2019)
Human iPSCs	Gene-edited cells with APOE4 alleles	ECM	APOE4 produces specific changes associated with AD	Model for studying the pathogenesis of AD and for evaluating more effective and personalized therapies	Yin et al. (2016)
Human iPSCs	Patient-derived cells with PSEN1 mutations	ECM	Beta-amyloid, neurofibrillary tangles and synaptic loss	Model for studying the pathogenesis of AD and for evaluating more effective and personalized therapies	Wang et al. (2017)
Human iPSCs	Patient-derived cells with PSEN1 mutations or Down’s Syndrom	ECM Orbital shaker	Beta-amyloid and neurofibrillary tangles	Model for studying the pathogenesis of AD and for evaluating more effective and personalized therapies	Gonzalez et al. (2018)
Human iPSCs	Sporadic AD patient-derived cells or APOE4 gene-edited cells	ECM Orbital shaker	REST-linked neuronal gene network dysregulation accelerates neuronal differentiation in AD	Model for studying the pathogenesis of AD and testing therapeutic interventions	Meyer et al. (2019)
Human iPSCs	Patient-derived cells with PSEN1 mutations	ECM	Beta-amyloid, inflammation, syndecan-3 and matrix changes	Model for studying the pathogenesis of AD and for evaluating more effective and personalized therapies	Yan et al. (2018b)

Organoids created from patient-derived iPSCs with familial AD mutations, such as PSEN1, as well as those derived from individuals with Down’s syndrome, have shown consistent production of beta-amyloid plaques and neurofibrillary tangles (Raja et al., 2016; Gonzalez et al., 2018). Additionally, APOE4 gene-edited iPSC-derived organoids have demonstrated distinct molecular and cellular alterations associated with this high-risk allele, further validating the role of APOE4 in AD pathogenesis and its utility in developing personalized therapies (Lin et al., 2018; Meyer et al., 2019).

Some studies have also incorporated orbital shakers and different ECMs enhance organoid maturation and structural organization. Notably, PSEN1 mutant organoids supported with Geltrex® exhibited not only Aβ and inflammatory changes but also modifications in matrix components such as syndecan-3, reflecting additional aspects of AD pathology (Yan Y. et al., 2018).

In conclusion, the application of iPSC-based brain organoids, particularly those genetically engineered to carry AD-related mutations or derived from patient cells, offers a robust and physiologically relevant platform to study disease mechanisms. By faithfully replicating core pathological features—such as Aβ deposition, tau aggregation, inflammation, and synaptic loss—these organoids enable the identification of early disease markers and facilitate the development of more effective, personalized therapeutic strategies.

#### 13.8.2 Amyotrophic lateral sclerosis

ALS is a neurodegenerative disease characterized by the progressive degeneration of upper and lower motor neurons, leading to widespread muscle weakness and, ultimately, respiratory failure. Several mechanisms are implicated in its pathogenesis, including excitotoxicity, mitochondrial dysfunction, oxidative stress, and the accumulation of proteins such as TDP-43 in the cytoplasm of affected neurons. However, the heterogeneity of the disease and variability in its progression have made it challenging to identify effective treatments (Ren et al., 2024). Additionally, genetic mutations, including C9orf72 expansions and TARDBP mutations, contribute to the heterogeneity of ALS, further complicating treatment development (Guo et al., 2024). The variability in disease progression and the lack of effective disease-modifying therapies highlight the urgent need for advanced models to study ALS pathology and identify therapeutic targets (Petersilie et al., 2024).

Various *in vitro* models have been employed to study ALS, including 2D cultures derived from induced iPSCs. These models have provided insights into neuronal excitability alterations and motor neuron degeneration (Bhargava et al., 2022). However, they present significant limitations as they fail to accurately replicate the 3D cytoarchitecture of the nervous system and the complex interactions between neurons and glial cells (Du et al., 2023).

To address the limitations of traditional ALS models, 3D organoid technology has emerged as a powerful platform that better mimics human neural architecture and cellular interactions. Derived primarily from human iPSCs, these models capture key pathological features of ALS, including synaptic hyperexcitability, motor neuron degeneration, and protein aggregation (Guo et al., 2024; Wang et al., 2022). They also allow for personalized disease modeling by incorporating mutations such as C9orf72 and TDP-43, which are commonly found in familial ALS cases (Guo et al., 2024; Nie et al., 2025; Casiraghi et al., 2025; de Majo et al., 2023). In addition, CRISPR-Cas9 gene-editing approaches have been successfully applied to generate or correct disease-associated mutations, demonstrating the potential of these models for testing therapeutic interventions (Nie et al., 2025; Meijboom et al., 2022).

Several specialized organoid types have been developed to explore specific aspects of ALS pathology (Table 2). Cortical organoids have been used to investigate early molecular changes such as synaptic hyperexcitability and protein aggregation, providing insight into neuron-glia interactions (Wang et al., 2022). Spinal cord organoids effectively model motor neuron degeneration and axonal loss, mimicking key features of ALS-related neurodegeneration (Guo et al., 2024). Motor organoids, derived through co-culture of neural and glial progenitors, are valuable tools to assess glial contribution to disease progression (Zhou et al., 2023; Taga et al., 2021; Workman et al., 2023).

**TABLE 2:** Summary of organoid models used in Amyotrophic lateral sclerosis research. The table summarizes general information regarding the cell source, induction method, support method, main findings obtained using each approach, potential applications, and corresponding references.

Disease: amyotrophic lateral sclerosis					
Cell source	Induction method	Support method	Key findings	Applications	References
iPSCs or ESCs	ALS patient-derived cells or genome-edited iPSCs	ECM	Synaptic hyperexcitability, protein aggregation	Analysis of cortical excitability, neuron-glia interaction	Wang et al. (2022)
iPSCs	Patterned differentiation to spinal motor neurons	ECM	Motor neuron degeneration, axonal loss	Modeling spinal pathology, ALS-related neurodegeneration	Guo et al. (2024)
iPSCs	Co-culture of neural and glial progenitors	ECM	Glial modulation of ALS phenotypes	Study of glial contribution to disease progression	Zhou et al. (2023), Taga et al. (2021), Workman et al. (2023)
iPSCs	Fusion of region-specific organoids	ECM or microfluidics	Impaired connectivity, motor network dysfunction	Exploring cortico-spinal interactions in ALS	(Andersen et al., 2020; Castillo Bautista and Sterneckert, 2022)
iPSCs	Co-differentiation with microglial progenitors	ECM	Neuroinflammation, reactive astrocytes, microglial imbalance	Studying inflammatory mechanisms in ALS	Hong et al. (2023)
iPSCs	Co-culture of skeletal muscle and spinal motor neurons	ECM	Functional NMJ formation, impaired neuromuscular connectivity	NMJ pathology in ALS, therapeutic screening	Kim et al. (2023)
Patient-derived iPSCs	Expansion mutation in C9orf72 gene	ECM	RNA foci, dipeptide repeats, neuronal stress	Familial ALS modeling, repeat-associated toxicity	Guo et al. (2024), Nie et al. (2025)
Genome-edited iPSCs	CRISPR/Cas9-based mutation	ECM	TDP-43 mislocalization and phosphorylation	Investigating TDP-43 proteinopathy in ALS	Casiraghi et al. (2025), De Majo et al. (2023)
C9orf72-mutant iPSCs	Gene correction using CRISPR/Cas9	ECM	Reversal of ALS pathology, restored cellular homeostasis	Validating genetic correction strategies	Nie et al. (2025), Meijboom et al. (2022)

Further refinement has led to the creation of hybrid organoids, combining cortical and spinal components, which allow researchers to examine cortico-spinal connectivity impairments—a hallmark of ALS motor network dysfunction (Andersen et al., 2020; Castillo Bautista and Sterneckert, 2022). Additionally, microglia-containing organoids show aspects of neuroinflammation and microglial imbalance, highlighting the role of immune responses in ALS pathogenesis (Hong et al., 2023). Neuromuscular organoids, which integrate skeletal muscle and spinal motor neurons, successfully model functional neuromuscular junction (NMJ) formation and are instrumental in evaluating therapies targeting NMJ integrity (Kim et al., 2023).

In conclusion, ALS organoids offer a physiologically relevant and patient-specific approach for studying disease mechanisms, identifying drug targets, and developing gene-editing therapies. The use of models incorporating ALS-linked mutations such as C9orf72 and TDP-43, as well as corrected variants through CRISPR-Cas9, further emphasizes their potential for advancing precision medicine in ALS (Guo et al., 2024; Nie et al., 2025; Casiraghi et al., 2025; De Majo et al., 2023; Meijboom et al., 2022).

#### 13.8.3 Parkinson’s disease

PD is a progressive neurodegenerative disorder characterized by the loss of dopaminergic neurons in the substantia nigra, leading to motor symptoms such as bradykinesia, rigidity, and resting tremors (Kim et al., 2019b; Jo et al., 2016). The etiology of PD is multifactorial, involving both genetic and environmental factors. Approximately 10% of cases are linked to genetic mutations in genes like SNCA, LRRK2, PINK1, PARK2, and GBA1, while the majority of cases are idiopathic (Kim et al., 2019b).

*In vitro* models have played a crucial role in understanding PD pathogenesis. The use of iPSCs has enabled the generation of patient-derived dopaminergic neurons, providing insights into key disease mechanisms such as α-synuclein aggregation, mitochondrial dysfunction, and oxidative stress (Kim et al., 2019b; Jo et al., 2016). However, traditional 2D cultures have limitations in replicating the complex 3D architecture of the human brain, leading to the development of 3D organoid models (Jo et al., 2016).

Brain organoids have become a transformative platform in PD research, offering human-specific models to investigate disease mechanisms with greater physiological relevance (Table 3). Among these, midbrain organoids derived from iPSCs recapitulate dopaminergic neuron development and neuromelanin production, enabling researchers to model key PD hallmarks such as α-synuclein aggregation and neuron loss (Kim et al., 2019b; Nguyen et al., 2011). Notably, genetically modified organoids carrying mutations in LRRK2 (G2019S), SNCA, or PINK1 have demonstrated mitochondrial dysfunction, oxidative stress, and impaired dopaminergic neurogenesis, making them ideal tools for investigating familial PD and evaluating potential therapies (Nguyen et al., 2011; Bolognin et al., 2019; Smits et al., 2019; Becerra-Calixto et al., 2023; Brown et al., 2021).

**TABLE 3:** Summary of organoid models used in Parkinson’s disease research. The table summarizes general information regarding the cell source, induction method, support method, main findings obtained using each approach, potential applications, and corresponding references.

Disease: Parkinson’s Disease					
Cell source	Induction method	Support method	Key findings	Applications	References
Human iPSCs	Induction of a G2019S mutation in LRRK2 with CRISPR/Cas9	ECM Orbital shaker	α-synuclein, TXNIP dysregulation, dopaminergic neurodegeneration	Model for understanding pathogenesis of PD and screening personalized therapies	Stachowiak et al. (2017)
Human iPSCs	Assembling striatal and cortical organoids derived from patients with a deletion in chromosome 22q13.3	ECM	Defects in calcium activity	Investigation of the cortico-striatal connectivity	Miura et al. (2020)
Human iPSCs	Midbrain organoids Toxic α-synuclein preformed fibrils	ECM	MLKL as a therapeutic target for reducing neuroinflammation and motor deficits in PD	Model for studying pathogenesis in PD and for evaluating potential therapies	Geng et al. (2023)
Human iPSCs	Patient-derived cells carrying the p.G2019S mutation in the LRRK2 gene	ECM	Oxidative stress response genes and α-synuclein protein	Model for identifying novel pharmacological agents and diagnostics	Nguyen et al. (2011)
Human iPSCs	Patient-derived cells carrying the LRRK2-G2019S mutation	ECM Organ-on-a-chip	DA neurons number and complexity loss Alteration in mitochondria morphology Treatment with LRRK2 inhibitor	Model for studying pathogenic mechanisms in PD and potential therapeutics	Bolognin et al. (2019)
Human IPScs	Patient-derived cells carrying the LRRK2-G2019S mutation	ECM	Decrease in the number and complexity of DA neurons Increase in FOXA2	Model for studying pathogenic mechanisms in PD and potential therapeutics	Smits et al. (2019)
Human IPScs	Patient-derived cells carrying the SNCA triplication	ECM	α-synuclein, Lewy bodies, loss of DA neurons and elevated apoptosis Increase in FOXA2	Model for studying pathogenic mechanisms in PD and therapeutic compounds	Becerra-Calixto et al. (2023)
Human iPSCs	PINK1-KO cells	Suspension Orbital shaker	Impeded DA neurogenesis	Model for studying pathogenesis in PD and future therapeutic approaches	Brown et al. (2021)
Human ESCs	Gen-edited cells carrying DNAJC6 mutations	ECM Orbital shaker	DA neuron degeneration, α-synuclein, and mitochondrial and lysosomal dysfunctions	Model for studying pathogenesi and assessing therapeutic interventions	Wulansari et al. (2021)
Human iPScs	Patient-derived cells carrying the GBA1 mutation	ECM Orbital shaker	Diminished GCase activity, defective complex I activity and α-synuclein	Model for studying pathogenesis in PD and developing therapeutic compounds	Baden et al. (2023)
Human iPSCs	Patient-derived cells carrying PRKN mutations	ECM	Astrocytic alteration and DA neurons loss	Model for studying pathogenic mechanisms in PD	Kano et al. (2020)
Human iPSCs	Treatment with 6-OHDA	ECM	DA neurons loss and neurite fragmentation	Model for studying PD and testing neurotoxic compounds	Monzel et al. (2020)
Human iPSCs	Treatment with MPTP	ECM	Massive DA neurons death	Platform for PD modeling and drug screening	Kwak et al. (2020)
Human iPSCs	Patient-derived cells carrying the A53T mutation	Organ-on-a-chip Gut-liver-cerebral organoid	Microbiome-associated short-chain fatty acids increase pathology-associated pathways	Model for studying the implication of the gut-liver brain axis and the A53T mutation in PD	Trapecar et al. (2021)
Human iPSCs	Patient-derived cells with DJ1 KO	Suspension	Impaired lysosomal proteolysis α-synuclein and astrocyte disfunction	Model for studying the pathogenesis in PD and uncovering potential therapeutic strategies	Morrone et al. (2024)

In addition to single-region models, advanced organoid systems now incorporate multiple brain regions. For example, cortico-striatal assembloids derived from patient cells with chromosomal deletions reveal defects in neuronal connectivity, aiding the study of circuit-level dysfunction in PD (Miura et al., 2020). Moreover, gut-brain assembloids using SNCA A53T patient-derived cells explore microbiome-derived influences on PD progression, highlighting the importance of the gut-liver-brain axis (Trapecar et al., 2021). These models expand our understanding of both central and peripheral contributors to PD pathology.

Environmental models using toxins such as MPTP and 6-OHDA have also been developed, inducing dopaminergic neuron death and neurite fragmentation. These models serve as robust tools for studying sporadic PD and screening neuroprotective agents (Monzel et al., 2020; Kwak et al., 2020). Additionally, organoids generated from GBA1, DNAJC6, PRKN, and DJ-1 mutant cells exhibit lysosomal dysfunction, mitochondrial damage, and astrocyte impairment—further supporting their use in modeling early-onset and atypical PD subtypes (Wulansari et al., 2021; Baden et al., 2023; Kano et al., 2020; Morrone et al., 2024).

Collectively, these PD-specific organoid systems offer unprecedented opportunities for drug discovery and precision medicine. CRISPR-Cas9 and organ-on-a-chip technologies have further enhanced the versatility of these models, enabling disease-specific modifications and improving physiological fidelity (Bolognin et al., 2019; Smits et al., 2019). As these tools evolve, they will continue to refine our understanding of PD pathogenesis and accelerate the development of targeted therapies.

#### 13.8.4 Multiple sclerosis

MS is a chronic autoimmune disease affecting the CNS. It is characterized by demyelination, neuroinflammation, and neurodegeneration, leading to motor and cognitive impairments (Czpakowska et al., 2024). The pathogenesis of MS remains complex, involving genetic, environmental, and immunological factors. Due to the limited understanding of its etiology, various *in vitro* models have been developed to study MS mechanisms and therapeutic targets (Engelhardt et al., 2022).

The prevalence of NDDs, including MS, is rapidly increasing as the aging population grows. However, current treatments only alleviate symptoms without stopping disease progression. The development of novel models using human-derived cells, such as brain organoids, is crucial for advancing our understanding of MS pathogenesis and drug discovery (Lei et al., 2024).

Traditionally, MS research has relied on *in vivo* models, particularly experimental autoimmune encephalomyelitis. However, *in vitro* models provide controlled environments to study specific cellular and molecular mechanisms (Wilhelm et al., 2011). These models include 2D cultures of neural and glial cells, as well as more advanced 3D cultures such as spheroids and organoids (Daviaud et al., 2023).

Recent research highlights the limitations of traditional models. Animal models do not fully replicate human genetic and cellular complexities, and 2D cultures lack the interactions found in the 3D brain environment. Organoids bridge this gap by providing a more physiologically relevant system that models the spatial structure and cellular interactions of the brain (Lei et al., 2024).

Organoid technology has become a pivotal tool in MS research, enabling the study of neurodegeneration, demyelination, and immune interactions in a human-relevant system (Table 4). Derived from iPSCs or ESCs, cerebral organoids recapitulate aspects of brain cytoarchitecture and region-specific development. Though they typically show low oligodendrocyte content, they have been used to study developmental alterations and the impact of inflammation on neural progenitors in MS (Daviaud et al., 2023; Wang, 2018).

**TABLE 4:** Summary of organoid models used in Multiple Sclerosis research. The table summarizes general information regarding the cell source, induction method, support method, main findings obtained using each approach, potential applications, and corresponding references.

Disease: multiple sclerosis					
Cell source	Induction method	Support method	Key findings	Applications	References
iPSCs or ESCs	Standard neural differentiation protocols	ECM or free-floating cultures	Exhibit diverse brain regions, low oligodendrocyte presence	Studying developmental abnormalities and brain region-specific effects of inflammation in MS	Daviaud et al. (2023), Wang (2018)
iPSCs o ESCs	Directed differentiation toward oligodendrocyte lineage	ECM	High oligodendrocyte content, capable of forming myelin	Modeling demyelination/remyelination processes; testing promyelinating therapies	Li and Shi (2020), Zeldich and Rajkumar (2024)
iPSCs from MS patients	Reprogramming and differentiation into neural/glial lineages	ECM, hydrogels, or scaffold-free	Exhibit genetic susceptibility and patient-specific immune phenotypes	Personalized medicine; studying patient-specific neuroimmune interactions	Li and Shi (2020)
iPSCs with multipotent differentiation	Co-culture of astrocytes, microglia, and oligodendrocytes	ECM or spheroid culture	Glia-glia crosstalk, neuroinflammation, and neuronal damage	Modeling inflammatory glial interactions and progression mechanisms in MS	Mrza et al. (2024), Stöberl et al. (2023)
iPSCs or human brain-derived cells	Differentiation into endothelial cells, astrocytes, pericytes	ECM or ECM-free	Blood-brain barrier dysfunction and immune cell infiltration	Studying vascular dysfunction, CNS immune access, and drug permeability in MS	Summers et al. (2024)
iPSCs differentiated into glial cells	Exposure to CSF from MS patients	Glia-enriched 3D cultures	Induction of reactive gliosis and neuronal damage via patient-derived factors	Modeling patient-specific inflammatory environments and immune-driven neurodegeneration	Fagiani et al. (2024)
Genetically modified iPSCs	Knock-out of NF155, a key axon-myelin adhesion protein	ECM	Axon-myelin disconnection and myelin instability	Investigating axon-myelin integrity and its disruption in MS pathogenesis	James et al. (2021)

To better address myelin-related pathology, oligodendrocyte-containing organoids have been developed using directed differentiation protocols. These models are capable of forming compact myelin and have been instrumental in studying demyelination/remyelination dynamics and screening promyelinating compounds (Li and Shi, 2020; Zeldich and Rajkumar, 2024). Further advancing the field, patient-derived iPSC organoids reflect genetic risk factors and immune phenotypes, offering personalized platforms for studying MS heterogeneity and tailoring treatments (Czpakowska et al., 2024).

More complex systems, such as co-cultured glial organoids, incorporate astrocytes, oligodendrocytes, and microglia to simulate the glial crosstalk that drives neuroinflammation and neurodegeneration in MS. These models allow researchers to investigate inflammatory mechanisms and glia-driven damage in three-dimensional environments (Mrza et al., 2024; Stöberl et al., 2023). Similarly, glia-enriched organoids exposed to CSF from MS patients show reactive gliosis and neuronal loss, making them a compelling model for studying the direct impact of patient-derived inflammatory factors (Fagiani et al., 2024).

Organoids mimicking the BBB are another essential advancement, integrating endothelial cells, astrocytes, and pericytes to evaluate BBB dysfunction—a hallmark of MS pathophysiology. These models help assess immune cell infiltration and screen therapeutic strategies targeting vascular integrity and drug permeability (Summers et al., 2024).

Finally, the creation of iPSC-derived myelinoids with neurofascin 155 (NF155) deficiency has shed light on axon-myelin interactions. These models highlight the role of NF155 in maintaining myelin stability and provide valuable insights into its contribution to MS-related myelin disruption (James et al., 2021).

Together, these diverse organoid systems offer new perspectives on MS pathology, from genetic susceptibility and immune involvement to remyelination potential and vascular contributions. Their continued evolution—through vascularization, gene editing, and multi-organoid assembly—promises to deepen our understanding of MS and accelerate the discovery of precision therapies.

## 14 Limitations and future advancements

As brain organoids are a relatively new technology, several challenges must be overcome to improve their effectiveness and reliability as models of the human brain. The primary issues include limited reproducibility and maturation, the formation of a necrotic core, and the absence of key brain characteristics such as gyrification, as well as essential non-neuronal cell types like microglia and oligodendrocytes.

A major challenge in organoid research is the lack of standardized protocols for reproducibility. Current methods often rely on poorly documented steps, require skilled personnel, and involve high costs, leading to variability across laboratories (Soldner and Jaenisch, 2018). The incorporation of diverse growth factors or nutritional components further complicates standardization, as each laboratory uses its own components (Kim et al., 2020). Commercial certified kits could minimize batch-to-batch variability. Simplifying protocols has shown promise, as evidenced by improvements in dorsal forebrain organoid generation (Velasco et al., 2019). Novel techniques like using micropillar arrays that enable the generation of contamination-free 3D cultures have improved neuronal differentiation and regionalization (Zhu et al., 2017). Hydrogels (Lancaster and Knoblich, 2014a) and 3D bioprinting (D’Antoni et al., 2023) are also emerging as alternatives to improve reproducibility and structural complexity. Moreover, the use of oncogenes for reprogramming iPSCs introduces risks, such as gene mutations, that may distort study outcomes, as they lead to genotype heterogeneity, resulting in diverse phenotypes (Halevy and Urbach, 2014). Culturing hPSCs in defined and xenogen-free conditions before detaching and seeding them to form EBs can enhance reproducibility and stem cell quality (Rothenbücher et al., 2021). Establishing accessible biobanks with clinical data for researchers could be a valuable approach, applicable to a variety of conditions, and form the foundation for future modeling strategies (Evangelisti et al., 2024). Variability can also be reduced by using genome-editing strategies for isogenic controls, considering polygenic risk scores (Soldner et al., 2009), and guiding regional fate with cytokines (Kim et al., 2020). These improvements could enhance reproducibility and translational value for disease modeling and drug testing. However, despite its transformative impact, CRISPR-Cas9 also faces challenges, including off-target effects, dependency on protospacer adjacent motif sequences, and immune responses to Cas9 components. Therefore, advances in engineering and delivery methods are essential to enhance its precision and applicability (Moon et al., 2019).

Cerebral organoids capture early stages of brain development, but they fail to form later structures, such as cortical plate layers. This limits their ability to study late-onset neurodegenerative stages such as synaptic loss or chronic gliosis. However, they provide a powerful platform to study early disease onset, including amyloid aggregation, mitochondrial stress, and neuroinflammatory responses (Raja et al., 2016). Efforts to induce maturation include using physiological glucose levels (Rocktäschel et al., 2019), telomerase inhibition (Vera et al., 2016), progerin overexpression (Miller et al., 2013) or removing antioxidant components from the culture (Kim et al., 2019b). Organoid fusion (Ahammed and Kalangi, 2024) and *in vivo* transplantation (Mansour et al., 2018; Revah et al., 2022) have also been shown to enhance maturity by exposing cultures to natural microenvironments. Genome-editing technologies could also be used to introduce mitochondrial dysfunctions associated with aging (Soldner et al., 2009). In addition to these approaches, direct reprogramming techniques, such as cellular transdifferentiation, offer the potential for age-preserved models by generating post-mitotic cells like neurons and glial cells, which could enhance aging studies in organoids. These advancements could enable the creation of multicellular, age-equivalent organoid models for studying aging and age-related diseases, overcoming some of the current limitations such as incomplete aging profiles and limited vascularization. Furthermore, by combining age-equivalent tissue models with heterochronic paradigms, future research could explore how aging processes interact with proliferative cells in 3D environments, opening new avenues for therapeutic interventions (Pitrez et al., 2024).

Beyond maturation issues, another critical limitation is the lack of vascularization, which leads to cell death in the core of the tissue, by restricting oxygen and nutrient delivery, hindering metabolite elimination, and disrupting cell signaling (Lancaster and Knoblich, 2014b). Therefore, many teams are working on different solutions. Coculturing organoids with endothelial cells, embedding them in ECMs or using HUVECs (Shi et al., 2020) fosters vascularization and enhances neurogenesis and maturation. Transplantation of organoids into animals (Mansour et al., 2018; Revah et al., 2022) and genetic engineering, such as using hETV2- a key transcription factor in vascular development-to induce vascular-like networks (Cakir et al., 2022), have also been successful in reducing hypoxia and necrosis. Other teams simply slice organoids to mitigate hypoxia (Choe et al., 2021), coculture vascularized brain organoids with choroid plexus organoids to produce cerebrospinal fluid and more complete vasculature (Pellegrini et al., 2020) Other solutions include spinning bioreactors, microfluidic devices (Lancaster and Knoblich, 2014a), ALI cultures (Cho et al., 2021), silk scaffolds (Sozzi et al., 2022), PCL scaffolds (Rothenbücher et al., 2021) and carbon fibers (Tejchman et al., 2020). All these advancements improve vascularization and nutrient delivery, creating more functional organoids for precise and cost-effective neurodegenerative disease research.

The lack of microglia, oligodendrocytes, and other essential cell types limits organoid utility for studying neuroinflammation and myelination. Introducing microglia or oligodendrocyte precursors into organoids has enabled the modeling of neuroinflammatory interactions and myelination processes (D’Antoni et al., 2023; López-Muguruza and Matute, 2023). Moreover, BBB organoids that include glial, vascular and neural cells may be a novel approach to model dysregulated neuroinflammatory processes implicated in NDDs such as MS(99,100).

Organoids lack cortical gyrification, a central developmental feature of the human brain, limiting their ability to successfully model human brain structure. Organ-on-a-chip systems (Karzbrun et al., 2018) and engineered flat brain organoids (Rothenbücher et al., 2021) may simulate mechanical and biological conditions driving the brain folding, offering insights into neocortical development and related disorders, and increasing the brain organoid’s relevance as a human brain model.

Lastly, the moral status of brain organoids requires consideration from multiple ethical perspectives. While current organoids are not sentient, ethical discussions aim to anticipate future challenges related to the possibility that brain organoids could 1 day exhibit consciousness-like features. The debate around brain organoids and consciousness arises because they might develop structures or functions similar to those of a developing human brain. If they reach a level comparable to a 20-week-old fetus, they could potentially have subjective experiences or suffer—even without feeling pain. This means they might have moral status, and research on them should be limited accordingly. Given the uncertainty, many argue that we should adopt a precautionary approach and treat potentially conscious organoids with ethical consideration. Categorizing brain organoids based on their maturation and interaction capabilities may help ensure ethical research (Koplin and Savulescu, 2019). Additionally, brain organoids offer a promising alternative to animal testing, aligning with ethical principles like the 3Rs (Replacement, Reduction, Refinement) to minimize animal use in neurobiological studies.

## 15 Concluding remarks and future directions

The development of brain organoids has revolutionized neuroscience by providing 3D models that reproduce some aspects of human brain complexity. Unlike traditional animal models, which often fail to fully reproduce human-specific neurophysiological processes due to species differences (Quadrato et al., 2016; Bjornson-Hooper et al., 2022), organoids offer a more physiologically relevant—though still simplified—representation of human brain development and disease mechanisms. Additionally, compared to 2D cultures, which lack the 3D cellular interactions and structural complexity required to capture key aspects of *in vivo* organization (Yang et al., 2023; Saraswathibhatla et al., 2023), organoids enable better cell-cell communication and microenvironment interactions. Compared to neurospheres, another 3D model, organoids offer superior complexity, replicating the organization, regional specification and cortical layering of the CNS, making them more suitable for studying complex neurodevelopmental processes (D’Antoni et al., 2023). Their ability to self-organize, generate diverse neuronal and glial cell populations (Clevers, 2016), and sustain long-term cultures (Quadrato et al., 2017; Trujillo et al., 2019) has significantly enhanced our understanding of neurodevelopmental and pathological processes, by capturing key cellular and molecular characteristics, facilitating the study of interactions between different cell types and assessing the impact of genetic and environmental factors on disease on-set (Choi et al., 2014; Raja et al., 2016; Kwart et al., 2019). Additionally, their use reduces reliance on animal experimentation, aligning with ethical principles.

Although some brain organoids are derived from ESCs, which raises additional ethical considerations, the use of iPSCs offers a valuable alternative, avoiding the moral implications associated with embryo use (De Wert and Mummery, 2003; Yang et al., 2023). Furthermore, as discussions about the potential for consciousness-like properties in brain organoids continue, it is expected that the scientific community will establish clear ethical and regulatory frameworks to guide future research in this area (Koplin and Savulescu, 2019), improving reproductivity and protocol harmonization across laboratories.

Organoids have demonstrated significant applications in biomedical research, including disease modeling, precision medicine, neurotoxicity evaluations, biomarker discovery, drug screening, gene editing, and biocompatibility testing (Corrò et al., 2020; Tang et al., 2022; Zhou et al., 2021; Romero et al., 2019). These models have enhanced our ability to study human-specific pathologies, test therapeutic compounds in a physiologically relevant environment, and develop personalized treatments based on patient-derived samples (Evangelisti et al., 2024; Tang et al., 2022).

One of the main advantages of organoids is their ability to model human diseases with greater fidelity than previous models. In the context of NDDs, the use of iPSCs is particularly advantageous, as they retain the phenotypic, genetic and epigenetic traits of the patient, allowing for personalized disease modeling by creating patient-specific and disease-specific cell lines (Halevy and Urbach, 2014; Dimos et al., 2008) and avoiding immune rejection after transplantation (Inoue et al., 2014). For conditions like PD and ALS, region-specific organoids provide the most effective approach, as they allow the generation of midbrain or spinal cord structures, which are the most affected regions in these diseases (Soldner et al., 2009; Andersen et al., 2020). By differentiating iPSCs into midbrain organoids for PD or spinal cord organoids for ALS, researchers can study region-specific neurodegeneration and screen targeted therapies more effectively (Jo et al., 2016; Faustino Martins et al., 2020).

For AD and MS, assembloids—fused organoids that interregional interactions—are the most effective models (D’Antoni et al., 2023; Qian et al., 2019). This is because AD involves widespread neurodegeneration across multiple brain regions, including the cortex and hippocampus, requiring interconnected organoids to simulate the complexity of disease mechanisms (Samarasinghe et al., 2021; Raja et al., 2016). Similarly, MS is characterized by immune system dysregulation and demyelination, which can be better studied in assembloids incorporating microglia and oligodendrocytes to model neuroinflammation and myelination processes (López-Muguruza and Matute, 2023; Madhavan et al., 2018).

Despite these benefits, organoid technology still faces multiple challenges. Before addressing these limitations, it is important to highlight additional benefits. Organoids provide a renewable and scalable source of human-relevant tissue, allowing long-term culture and high-throughput screening (Drost and Clevers, 2018). They also enable the study of patient-specific genetic mutations, the testing of gene-editing therapies like CRISPR-Cas9, and the development of patient-derived biobanks for future research (Evangelisti et al., 2024; Zhou et al., 2021; Li G. et al., 2022). In addition, generating assembloids (D’Antoni et al., 2023; Yang et al., 2023; Qian et al., 2019) and incorporating microglia (Cakir et al., 2022) and myelin-producing cells (López-Muguruza and Matute, 2023) have significantly improved the biological relevance of these models.

Current limitations of organoids include the lack of vascularization, leading to cell death in deeper layers due to insufficient nutrient and oxygen supply (Lancaster and Knoblich, 2014b), and limited neuronal maturation, which affects the modeling of late-onset diseases like AD and PD (Ahammed and Kalangi, 2024). For this last reason, the primary strength of organoids lies in their capacity to reproduce early-stage neurodegeneration, making them highly relevant for studying early on-set mechanisms, as it has been evidenced in PD (Morrone et al., 2024) and ALS brain organoids (Wang et al., 2022).

Strategies to address these issues involve organoid-on-a-chip systems with microfluidics for better vascularization and nutrient exchange (Cho et al., 2021; Castiglione et al., 2022), as well as maturation techniques such as adjusting glucose levels (Rocktäschel et al., 2019), telomerase inhibition (Vera et al., 2016), progerin overexpression (Miller et al., 2013), and reducing antioxidants (Kim et al., 2019b). Additionally, organoid fusion (Ahammed and Kalangi, 2024), *in vivo* transplantation (Mansour et al., 2018; Revah et al., 2022), and genome-editing for mitochondrial dysfunctions (Soldner et al., 2009) have been used to mimic aging processes. Co-culturing with endothelial cells (Lancaster and Knoblich, 2012; Pham et al., 2018; Shi et al., 2020), spinning bioreactors (Lancaster and Knoblich, 2014a), ALI cultures (Cho et al., 2021), scaffolds (Sozzi et al., 2022; Rothenbücher et al., 2021), and hydrogels (Rossi et al., 2018; Ranga et al., 2014) improve oxygen diffusion and support the growth of larger, more mature organoids. Standardization remains a challenge due to variability between cultures, but efforts to address this include simplified differentiation protocols (Velasco et al., 2019), automation (Evangelisti et al., 2024; Zhu et al., 2017), and bioengineering approaches (Soldner et al., 2009) to improve reproducibility and scalability.

In conclusion, brain organoids represent a powerful and continuously evolving tool for studying neuronal biology and nervous system diseases. While challenges remain, ongoing technological innovations—including organoid-on-a-chip systems, bioreactors, gene editing techniques and standardized protocols—suggest that these models could play a key role in translational medicine, bridging the gap between preclinical research and effective clinical applications (Yang et al., 2023; Qian et al., 2019; Corrò et al., 2020). Furthermore, integrating organoids with artificial intelligence and omics technologies could facilitate biomarkers identification and the development of personalized therapies for NDDs (Tang et al., 2022; Romero et al., 2019). The establishment of organoid biobanks would also expand access to these models for research and preclinical testing, fostering advancements in precision medicine (Evangelisti et al., 2024; Zhou et al., 2021).
